# A Novel Polymersome Nanocarrier Promotes Anti‐Tumour Immunity by Improved Priming of CD8 ^+^ T Cells

**DOI:** 10.1111/imm.13903

**Published:** 2025-01-28

**Authors:** Regine J. Dress, William W. Ho, Victor Ho, Jian Hang Lam, Fabien M. Décaillot, Gaurav Sinsinbar, Jenetta Soo, Gowshika Rengasamy, Amit Kumar Khan, Thomas Andrew Cornell, Teck Wan Chia, Shrinivas Venkataraman, Madhavan Nallani, Florent Ginhoux

**Affiliations:** ^1^ Singapore Immunology Network (SIgN), A*STAR Singapore Singapore; ^2^ Institute of Systems Immunology, Hamburg Center for Translational Immunology (HCTI) University Medical Center Hamburg‐Eppendorf Hamburg Germany; ^3^ ACM Biolabs Pte Ltd Singapore Singapore; ^4^ Sapreme Development B.V Bilthoven The Netherlands; ^5^ Unilever Industries Private Limited Singapore; ^6^ Gustave Roussy Cancer Campus Villejuif France; ^7^ Institut National de la Santé et de la Recherche Médicale (INSERM) U1015 Equipe Labellisée—Ligue Nationale Contre le Cancer Villejuif France; ^8^ Shanghai Institute of Immunology Shanghai Jiao Tong University School of Medicine Shanghai China

**Keywords:** ACM, Cancer, dendritic cells, melanoma, polymersome, T cell, therapy, vaccine

## Abstract

Cancer is one of the leading causes of death worldwide. In recent years, immune checkpoint inhibitor therapies, in addition to standard immuno‐ or chemotherapy and surgical approaches, have massively improved the outcome for cancer patients. However, these therapies have their limitations and improved strategies, including access to reliable cancer vaccines, are needed. Here, we describe the use of self‐assembling artificial cell membrane (ACM) polymersomes to deliver tumour‐specific peptides to trigger sustainable and efficient anti‐tumour immune responses. We found that ACM polymersomes were highly efficient in targeting and activating mononuclear phagocytes (MNP) including dendritic cells (DC), while providing long‐term reservoirs of antigens for continued immune cell priming. Subcutaneous injection of ACM‐encapsulated tumour‐antigen‐peptides into tumour‐bearing mice resulted in improved priming of CD8^+^ T cells and increased generation of tumour‐antigen‐peptide specific CD8^+^ effector T cells. Prophylactic and therapeutic immunisation with ACM‐encapsulated peptides resulted in changes to the MNP composition in the tumour microenvironment, tumour regression and improved survival of immunised mice. Combining anti‐PD‐1 immune checkpoint inhibitor therapy with ACM polymersome peptide delivery further boosted anti‐tumour immunity. Our results show that ACM polymersome nanocarriers efficiently instruct anti‐tumour immune responses offering a promising new approach for vaccination and cancer immunotherapy.

**Trial Registration:** NCT05385991

## Graphical abstract

Here, we show that subcutaneous injection of novel artificial cell membrane (ACM) polymersomes loaded with tumour‐specific antigens or peptides, such as OVA, results in efficient anti‐tumour immune responses. In both, a therapeutic and a prophylactic model, we found that ACMs were efficiently taken up by antigen presenting cells, such as dendritic cells, which then primed naïve T cells for specific targeting of tumour cells in B16‐OVA and B16F10 mouse melanoma models. This was accompanied by significant expansion of tumour‐specific and memory T cells, drastic reduction of tumour growth and increased survival of ACM‐injected mice over non‐ACM‐injected controls.

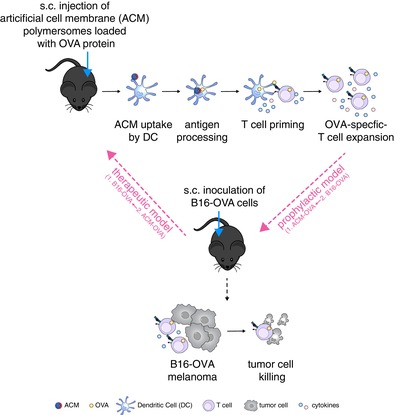

## 1 Introduction

Despite major advances in modern medicine, cancer is a leading cause of death among humans worldwide. Depending on the type of cancer, the stage or rate of tumour progression, disease severity and prognosis drastically vary, determining quality of life and survival chances of each individual patient. The development of immune checkpoint inhibitor therapies in recent years [1, 2, 3, 4, 5], in addition to chemotherapy and surgical approaches, has drastically improved prognosis, quality of life, and survival of cancer patients [4, 6]. Despite this, a permanent cure or complete prevention for every type of cancer remains not achieved. Thus, more research is needed to further our understanding of this multifaceted disease and to move forward with novel therapy and vaccination approaches.

Successful immunisation is achieved by efficient priming of innate and adaptive immune cells and by engagement and generation of antigen‐specific effector immune cells. These cells are key to modulating the tumour microenvironment (TME) towards tumour regression, and further to prevent metastatic and severe, late‐stage cancers [7, 8, 9]. Most vaccines currently available are designed for infectious diseases, with few vaccines specific for cancer‐inducing viruses, such as human papilloma virus [10] or Epstein–Barr Virus (EBV) [11]. These vaccines frequently utilise live attenuated or inactivated pathogens as antigens and are highly efficient in activating antigen‐presenting dendritic cells (DC). Activated DC then prime and instruct the proliferation of antigen‐specific effector and memory T and B cells, leading to long‐lasting immunity [12, 13, 14, 15]. Effector T cells, which can kill tumour cells in an antigen‐specific manner, are key to the overall anti‐tumour responses and to tumour regression [16]. In addition, successful cancer vaccines and therapies not only need to promote the proliferation of tumour‐specific T cells but also need to facilitate the modulation of the TME, away from an immunosuppressive towards a rather tumour‐suppressive environment. Newly developed mRNA vaccines or synthetic peptide vaccines have the potential to induce and activate immune cells in a more targeted and controlled manner, as compared to previous vaccine designs [17]. However, most synthetic peptides are not as immunogenic on their own, as compared to attenuated pathogens, and require adjuvants, such as toll‐like receptor (TLR) stimulating agents, in order to improve uptake by and activation of immune cells. Packaging of synthetic peptides or epitopes within vesicles or encapsulation within lipid membranes or nanoparticles are promising new approaches to enhance their immunogenicity and to potentially harness the patient's own immune system for cancer immunotherapy [18, 19, 20].

We previously designed self‐assembling artificial cell membrane (ACM) polymersomes and demonstrated that encapsulating proteins within these ACM polymersomes vastly improved their immunogenicity [19]. ACM polymersomes structurally are made up by amphiphilic block co‐polymers comprised of polybutadiene‐b‐polyethylene oxide (PBD‐PEO) and 1,2‐Dioleoyl‐3‐trimethylammonium propane (DOTAP), a cationic lipid [21]. Amphiphilic block co‐polymers such as PBD‐PEO are designed to undergo self‐assembly into thermodynamically stable vesicular structures. In such a vesicular structure, the hydrophobic PBD block assembles in the middle of the bilayer, minimising its interaction with the surrounding water, while leaving the water‐soluble PEO block exposed to the outside. During this self‐assembly process solutes can be encapsulated within the vesicular cavity. This ability to compartmentalise antigens and adjuvants, while maintaining their stability, renders these ACM polymersomes very attractive as novel nanocarriers for vaccine and therapy delivery [18, 22]. In a previous study in an infectious disease setting [19], we found that antigen‐loaded polymersomes efficiently targeted murine and human DCs.

Here, we employed ACM polymersomes to deliver tumour‐specific peptides to trigger sustainable and efficient anti‐tumour immune responses. We found that ACM polymersomes were efficiently taken up by mononuclear phagocytes (MNP), resulting in MNP activation and migration. Further, ACM polymersomes provided long‐term reservoirs of antigens allowing for continuous priming of immune cell. Mice challenged with B16‐OVA or B16F10 melanoma cells showed improved survival, tumour control and regression, as well as increased frequencies of tumour‐specific CD8^+^ effector T cells, when immunised with ACM‐encapsulated tumour‐antigen‐peptides. Additionally, combining anti‐PD‐1 immune checkpoint inhibitor therapy with tumour‐peptide delivery within ACM polymersomes further improved the anti‐tumour responses. Overall, we here demonstrate that ACM polymersome nanocarriers efficiently target antigen‐presenting cells and promote the generation of tumour‐specific CD8^+^ effector T cells, resulting in effective anti‐tumour immune responses, tumour regression and prolonged survival of mice. We here propose ACM polymersomes as promising new nanocarriers in the context of cancer immunotherapy and vaccination.

## 2 Results

### 2.1 Design and Physicochemical Characterisation of ACM Polymersomes as Stable Nanocarriers for Immunogenic Peptides

We previously developed artificial cell membrane ACM‐based polymersomes [19]. These are self‐assembling vesicles built from an amphiphilic block co‐polymer comprising of polybutadiene‐b‐polyethylene oxide (PBD‐PEO) and the cationic lipid 1,2‐dioleoyl‐3‐trimethylammonium‐propane (DOTAP). The advantage here is, that during self‐assembly solutes can be packaged within these vesicles, resulting in potent nanocarriers. The use of ACM polymersomes to deliver SARS‐CoV‐2 spike protein and CpG adjuvant, in the context of a COVID‐19 vaccine, has been reported earlier ([19]; ACS Nano, 2022). Here, we investigated the use of polymersomes of similar composition to deliver other APIs (see Table S1. for physicochemical characteristics of the different formulations). Fabrication of ACM‐formulations was based on the thin film rehydration method (Figure S1A) or co‐solvent method (Figure S1B).

**FIGURE 1:**
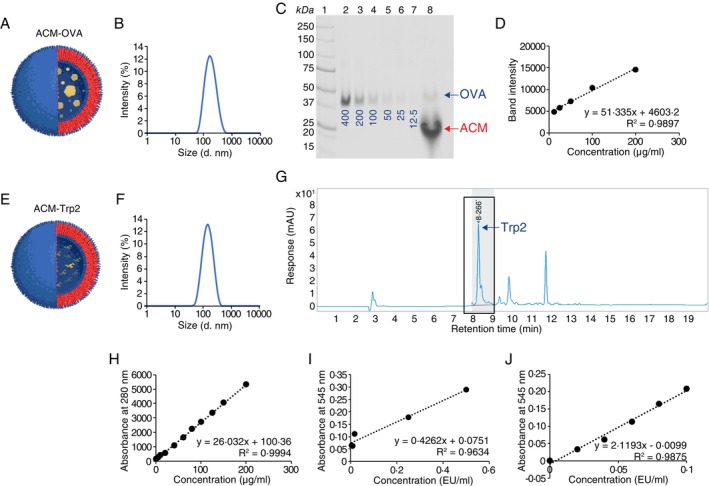
Physicochemical characterisation of ACM‐OVA and ACM‐Trp2. (A) Schematic illustration of ACM‐OVA. OVA protein (represented as yellow spheres) is encapsulated within the cavity of an ACM polymersome (represented as a bi‐layered membrane) vesicle. (B) Hydrodynamic diameter of ACM‐OVA as measured by dynamic light scattering (DLS) measurement. (C) Analyses of encapsulated OVA by SDS‐PAGE and SYPRO Ruby total protein stain. Lane 1 shows the Precision Plus Protein Standards. Lanes 2–7: Contain the purified OVA standards, loaded protein quantities (in μg) are indicated accordingly. Lane 8 was loaded with ACM‐OVA. ACM‐OVA polymersomes were lysed with Triton‐X 100 to release OVA prior loading. Positions of OVA (43 kDa) and disrupted polymersomes are indicated. (D) Standard curve for the quantification of encapsulated OVA concentrations as measured by densitometry. (E) Schematic illustration of ACM‐Trp2. Trp2 protein (represented as yellow bands) is encapsulated within the cavity of an ACM polymersome (represented as a bi‐layered membrane) vesicle. (F) DLS measurement of the hydrodynamic diameter of ACM‐Trp2. (G) Analysis of Trp2 by HPLC. Encapsulated peptide was released by detergent lysis and compared against purified peptide standards. The peak corresponding to Trp2 is indicated (retention time: 8.27 min). (H) Standard curve for quantification of encapsulated Trp2 concentrations as measured by HPLC. (I) Standard curve for quantification of associated endotoxin concentrations measured using the HEK‐Blue LPS Detection Kit. (J) Standard curve for quantification of associated endotoxin concentrations measured using the Chromogenic LAL Assay.

To test whether ACM polymersome nanocarriers can be used for cancer vaccination or therapy purposes, we first encapsulated Ovalbumin (OVA) within the vesicular cavity of ACM (Figure 1A). ACM polymersomes were prepared according to our previously described protocol [19] and the average polymersome size was measured using Dynamic light scattering (DLS) measurements. We found that OVA‐encapsulating ACM polymersomes (ACM‐OVA) followed a unimodal intensity‐weighted distribution with a mean *z*‐average hydrodynamic diameter of 162.0 nm and a polydispersity index (PDI) of ≤ 0.160 (Figure 1B). To quantify the amount of encapsulated OVA, ACM‐OVA polymersome vesicles were disrupted using Triton‐X 100 and the resulting lysate was analysed by SDS‐PAGE, alongside a series of purified OVA standards (ranging from 12.5 to 400 μg/mL) (Figure 1C). We then visualised the protein bands using SYPRO Ruby protein blot stain, performed densitometry measurements of each band and generated a standard curve, from which the concentration of ACM‐encapsulated OVA was established to be at 170 μg/mL (Figure 1C,D).

Upon this initial successful encapsulation of OVA, we decided to next encapsulate a peptide sequence which would allow us to test the immunogenicity of ACM polymersome nanocarriers in specific disease settings. Here, we chose a peptide (SVYDFFVWL) from the tyrosinase related protein‐2 (Trp2) protein, specific to murine B16 melanoma (Figure 1E). ACM polymersomes encapsulating Trp2 peptide (ACM‐Trp2) were prepared according to our previously described protocol [19] and DLS measurements showed ACM‐Trp2 formulations to follow unimodal intensity‐weighted distributions with a mean *z*‐average hydrodynamic diameter of 129.9 nm and a PDI of ≤ 0.160 (Figure 1E,F). For ACM‐Trp2, the encapsulated peptide was quantified using high‐performance liquid chromatography (HPLC). We established a retention time of 8.27 min for a purified Trp2, allowing us to clearly identify the Trp2 peak among the different molecular species within the ACM lysate (Figure 1G). Based on area under curve (AUC) measurements from the HPLC standards, we established a concentration of 190.6 μg/mL for the Trp2 peptide encapsulated within ACM polymersomes (Figure 1H).

To properly assess whether ACM polymersome nanocarriers can efficiently trigger immune responses, it is crucial to avoid contamination of the ACM formulations with other agents, such as bacterial lipopolysaccharides (LPS), that could activate for example, dendritic cells (DC) and influence the experimental outcome. Thus, for each formulation we determined the associated endotoxin concentration either using the HEK‐Blue LPS Detection Kit or the Chromogenic LAL Assay (Figure 1I,J). We confirmed that neither ACM‐OVA nor ACM‐Trp2 nanocarriers were contaminated by LPS, with LPS concentrations determined to be < 0.2 EU/ml, for either formulation (Figure 1I,J).

In more detail, ACM formulations were fabricated in a cleanroom and checked for endotoxin contamination using the HEK‐Blue LPS Detection Kit. Formulations were released for in vivo work only if they met the requirement of < 0.2 EU/mL LPS to mitigate non‐specific immune activation. We have also evaluated the reactogenicity of the empty ACM nanocarrier to determine its contribution to the immune response. Accordingly, human peripheral blood mononuclear cells (PBMCs) from five healthy donors were treated 2 days with 15 μg/mL of CpG encapsulated within ACM polymersomes or the empty vesicles alone. ACM‐CpG induced a broad range of cytokines and soluble mediators, including IL‐6, IFNγ, TNFα, IL‐10 and IL‐1RA, that were secreted into the culture supernatant (Figure S1C). In contrast, empty polymersomes triggered little to no response. These results indicate that polymersomes are poorly reactogenic and the observed immune response was predominantly driven by the encapsulated API.

Hence, we here successfully generated ACM polymersome nanocarriers that we then tested in different cancer vaccine and therapy strategies.

### 2.2 ACM Nanocarriers Engage Immune Cells Over Sustained Periods of Time

**FIGURE 2:**
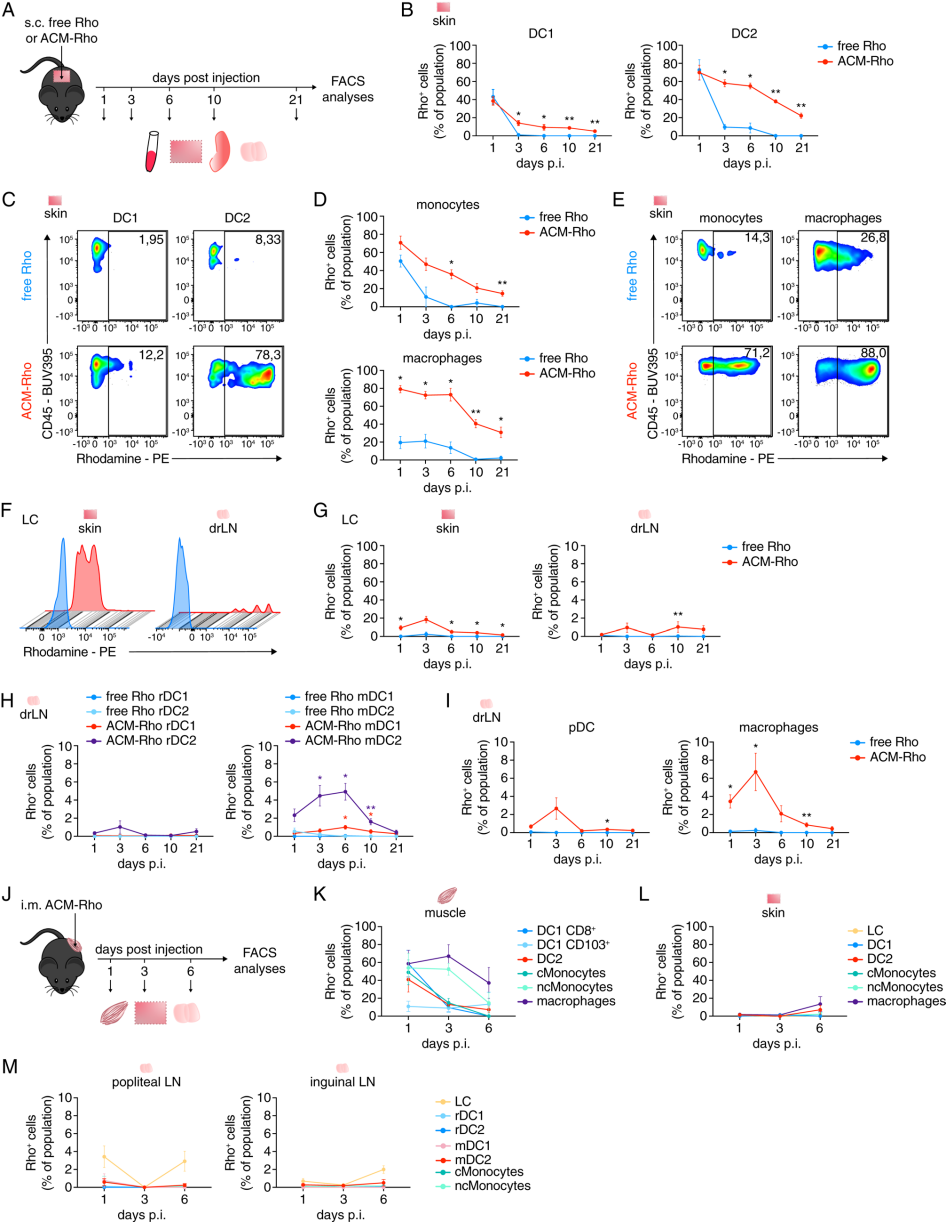
ACM polymersomes are efficiently phagocytosed and release their contents over a period of time. (A) Illustration of ACM‐Rhodamine (ACM‐Rho) injection protocol and time course. ACM‐Rho or free Rhodamine (free Rho) was injected subcutaneously (s.c.) into naïve wildtype mice and blood, skin, spleen and/or skin‐draining lymph nodes (drLN) were analysed by flow cytometry at indicated timepoints, ranging from 1 to 21 days post injection. (B) Graphs and (C) flow cytometry plots for day 3 p.i. showing the percentage of Rho^+^ DC1 and DC2 in the skin post s.c. injection of ACM‐Rho or free Rho. (D) Graphs showing the percentage of Rho^+^ monocytes and macrophages in the skin post s.c. injection of ACM‐Rho or free Rho. (E) Flow cytometry plots showing Rho signals in skin monocytes and macrophages on day 3 post s.c. injection. (F) Histograms showing Rho expression by LC in the skin 3 days post s.c. injection. (G) Graphs showing the percentage of Rho^+^ LC in the skin and drLN over time, post s.c. injection of ACM‐Rho or free Rho. (H) Percentage of Rho^+^ migratory and resident DC1 and DC2 in the drLN post s.c. injection of ACM‐Rho or free Rho. (I) Graphs showing the percentage of Rho^+^ pDC and macrophages in the drLN post s.c. injection. (J) Scheme illustrating the intramuscular (i.m.) injection protocol of ACM‐Rho into naïve wildtype mice. Injection‐side‐ drLN, skin and muscle tissue were analysed by flow cytometry at indicated timepoints. (K) Graphs showing the percentage of Rho^+^ DC and monocyte populations in the muscle post i.m. injection of ACM‐Rho. (L) Graphs showing the percentage of Rho^+^ DC and monocyte populations in the skin post i.m. injection of ACM‐Rho. (M) Graphs showing the percentage of Rho^+^ DC and monocyte populations in the drLN post i.m. injection of ACM‐Rho. (*n* = 3 to 5 mice per group, one to five independent experiments; data shown as mean ± SEM). Statistical analysis was done with unpaired two‐tailed *t* test. *p < 0.05; **p < 0.01; ***p < 0.001 and ****p < 0.0001.

Since successful vaccines and therapies rely on adequate immune activation, we first analysed the mechanisms by which ACM polymersomes may act on immune cells. To this purpose we subcutaneously (s.c.) injected C57BL/6 wildtype mice with the non‐toxic fluorescent dye Rhodamine (Rho) or Rho labelled ACM polymersomes (ACM‐Rho) and analysed persistence and uptake of Rho versus ACM‐Rho by different immune cells. Cells from the blood, skin, skin‐draining inguinal lymph nodes (drLN) close to the injection side, and spleen were analysed by flow cytometry at different time points post injection (p.i.) over a period of 3 weeks (Figure 2A). We observed that ACM‐Rho polymersomes were maintained long‐term within the subcutaneous compartment, with Rho dye still visible up to at least 140 days p.i., while free Rho could no longer be visually detected shortly after injection (Figure S2A).

DC are cells of the innate immune system highly specialised in phagocytosing and presenting antigens and priming of effector functions of naïve CD8^+^ and CD4^+^ T cells [13]. Among the different subsets of DC, DC1, which are specialised in cross‐presentation of antigens, have been increasingly recognised as promising targets for cancer therapy [9, 23, 24, 25]. Thus, we first focused our analyses on tissue DC, assuming they should be highly capable of phagocytosing ACM‐Rho polymersomes. In the skin both, DC1 and DC2 subsets, where highly Rho‐labelled, with 40% of DC1 and 70% of DC2 being Rho^+^, on day one p.i. of either free Rho or ACM‐Rho (Figure 2B). Frequencies of Rho^+^ skin DCs drastically reduced in mice injected with free Rho from day three p.i. onwards, while ~60% of DC2 and ~ 10% of DC1 remained Rho^+^ after ACM‐Rho injection, for up to 3 weeks p.i. (Figures 2B,C and S2B). Monocytes were highly labelled by free Rho and by ACM‐Rho on day one p.i. (50% and 70% respectively). Continued labeling of monocytes over time was only observed in mice injected with ACM‐Rho but not free Rho (Figure 2D,E). We observed similar kinetics for macrophages (Figures 2D,E and S2B), including Langerhans cells (LCs), which preferentially phagocytosed ACM‐Rho over free Rho, resulting in almost 20% Rho^+^ skin LCs on day three p.i. of ACM‐Rho (Figure 2F,G).

While frequencies of Rho^+^ skin LC decreased over time, there appeared to be a constant influx of Rho^+^ LCs to the drLN from day three p.i. onwards (Figure 2F,G). In line with this, we found Rho^+^ migratory DC2 (mDC) and mDC1 (on day 6 p.i.) in the drLN of mice injected with ACM‐Rho, but not of mice injected with free Rho or in the resident DC (rDC) populations (Figure 2H). ACM‐Rho‐uptake by plasmacytoid DC (pDC) was elevated, though not significantly, on day three p.i. (Figure 2I). At this timepoint we also detected significant uptake of ACM‐Rho in macrophages in the drLN, with some macrophages maintaining Rho‐signals till at least 10 days p.i. (Figure 2I). Further, we could not detect any Rho signal in the blood or spleen of mice injected with free Rho or ACM‐Rho, suggesting a locally contained immune response (Figure S2C).

To test whether multiple ACM polymersomes could be phagocytosed by the same cell, we generated ACM encapsulating green‐fluorescent DQ plus OVA (ACM‐DQ‐OVA). Mice were co‐injected s.c. with ACM‐Rho and ACM‐DQ‐OVA and immune cells of the skin and drLN were analysed for the presence of Rho^+^DQ^+^ cells by flow cytometry, as before. We found that 10% to 40% of skin DC2, monocytes or macrophages, but not DC1 or LC, were positive for Rho and DQ on days one to three p.i., suggesting these cells were able to phagocytose multiple ACM polymersomes at the same time. As we did not observe Rho^+^DQ^+^ cells in the drLN this, however, was not reflected by a migration of these cells to the drLN (Figure S2D).

Further, we ex vivo treated human PBMCs with increasing concentrations of ACM loaded with fluorescent rhodamine dye (ACM‐Rho), or fluorescent cyanine5 dye (ACM‐Cy5), or a mixture of both, ACM‐Rho plus ACM‐Cy5 for 3 h. Uptake of both ACMs by different subsets of monocytes (classical, intermediate and non‐classical) and DCs (myeloid mDC and pDC; Figure S3A) was then analysed. We found, that each cell type showed a dose‐dependent increase in fluorescence intensity in the PE or APC channel after treatment with ACM‐Rho or ACM‐Cy5, respectively. Importantly, each cell type treated with a 1:1 mix of ACM‐Rho and ACM‐Cy5 became double positive for both signals in a dose‐dependent manner (Figure S3B). Altogether, these results confirmed simultaneous uptake of different ACMs bearing different cargo by the same antigen presenting cell. These data demonstrate the possibility to co‐administer multiple ACM polymersome nanocarriers containing proteins, or perhaps vaccines and adjuvants, with a single injection.

To address whether the site of injection affects the uptake of ACM polymersomes by antigen presenting cells (APC), we repeated the ACM‐Rho protocol using intramuscular (i.m.) injections this time. (Figure 2J). Myeloid cells in the muscle were labelled most efficiently on days one and three p.i., with ~60% of macrophages, > 50% of monocytes, and ~40% of DC2 being Rho^+^ (Figure 2K). However, in the skin and draining LN, most cells had not taken up any Rho with only few Rho^+^ DC2, monocytes and macrophages, including LC, detected between days one to six p.i. (2%–20% per population, not significant; Figure 2L,M). Overall, both s.c. and i.m. injection efficiently targeted APC, but only s.c. injection appeared to result in a local Rho‐antigen reservoir allowing for continued exposure of APC to ACM polymersomes/antigens.

Collectively, we found that encapsulating Rho into ACM polymersomes vastly improved Rho half‐life within the skin compartment and uptake by APC, especially by DC and macrophage populations in the skin and skin drLN, rendering ACM polymersome nanocarriers promising candidates for novel vaccine and therapy delivery strategies.

### 2.3 Immunisation With ACM Polymersome Nanocarriers Minimises Tumour Burden and Boosts Expansion of CD8 ^+^ Effector T Cells in Vaccinated Mice

**FIGURE 3:**
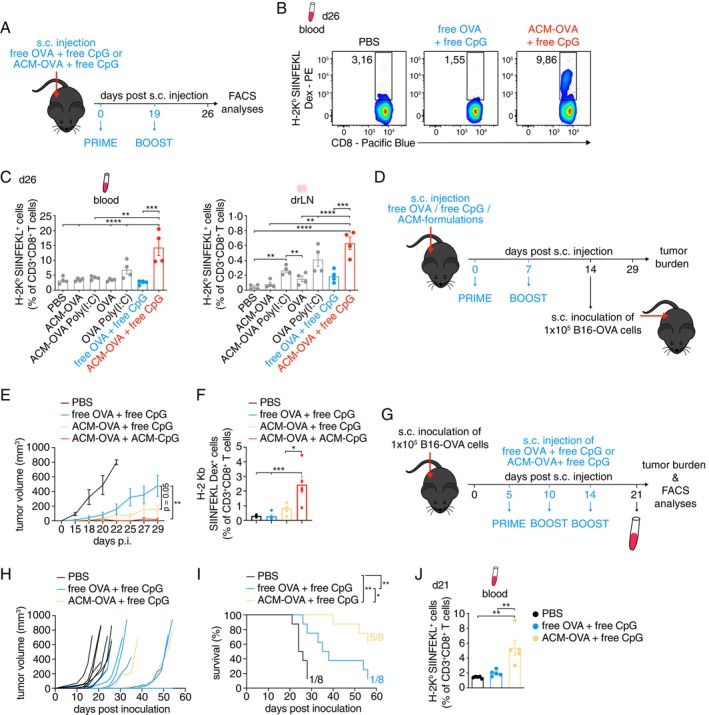
Vaccination with ACM‐OVA plus free CpG efficiently generates H‐2K^b^‐SIINFEKL‐specific CD8^+^ T cells preventing the expansion of B16‐OVA cancer cells. (A) Scheme of the injection protocol. Mice were s.c. injected with free OVA + free CpG or ACM‐OVA + free CpG, following a prime (day 0) and boost (day 19) regime, before analyses of blood by flow cytometry. (B) FACS plots showing the frequency of H‐2K^b^‐SIINFEKL‐specific CD8^+^ T cells in the blood on day 26 post prime immunisation. (C) Graphs show the frequency of H‐2K^b^‐SIINFEKL MHCI‐peptide complex^+^ CD8^+^ T cells in the blood and inguinal drLN on day 26 post prime immunisation. (*n* = 4). (D) Scheme of the vaccination protocol. Mice were s.c. injected with free OVA + free CpG or ACM‐OVA + free CpG or ACM‐OVA + ACM‐CpG on day 0 (prime) and day 7 (boost), before injection of 1 × 10^5^ B16‐OVA cancer cells into the right hind flank on day 14. (E) Average volume of B16‐OVA tumours in vaccinated vs. PBS injected wildtype mice. (*n* = 8). (F) Frequency of H‐2K^b^‐SIINFEKL‐specific CD8^+^ T cells in the blood on day 20 post prime immunisation. (G) Scheme of the therapeutic protocol. Mice were s.c. inoculated with 1 × 10^5^ B16‐OVA cancer cells into the right hind flank on day 0, followed by s.c. injection with free OVA plus + CpG or ACM‐OVA + free CpG on day 5 (prime) and days 10 and 14 (boosts). Blood was drawn on day 21 and tumour burden was monitored over a period of < 60 days. (H) Volume of B16‐OVA tumours in therapeutically injected mice versus PBS injected wildtype mice. (*n* = 8). (I) Curve shows the average survival of mice injected with free OVA plus + CpG or ACM‐OVA + free CpG or PBS post inoculation with B16‐OVA cancer cells. (*n* = 8). (J) Frequency of H‐2K^b^‐SIINFEKL MHCI‐peptide complex^+^ CD8^+^ T cells in the blood on day 21 post inoculation. (*n* = 5). Data are shown as Mean ± SEM. Statistical analysis was done with Two‐way ANOVA plus Turkey post‐test or unpaired two‐tailed *t* test. *p < 0.05; **p < 0.01; ***p < 0.001 and ****p < 0.0001.

Next, we wanted to test the potential of ACM polymersomes as vaccine carriers. Here, we employed the B16‐OVA melanoma mouse model, a well‐established cancer immunotherapy murine model. In short, in this model cancer cells express the model OVA peptide antigen (SIINFEKL), thus facilitating strong anti‐tumour responses and allowing for easy identification of cancer‐specific CD8^+^ effector T cells. To further strengthen the immune response elicited by ACM polymersomes, we co‐administered OVA with the toll‐like receptor 9 (TLR9) agonist CpG or TLR3 agonist Poly(I:C), which both aid in the activation of DC [26, 27]. In a first trial, C57BL/6 mice were injected with free OVA + free CpG or ACM‐OVA + free CpG, following a prime (day 0) and boost (day 19) regime (Figure 3A). We found that ACM‐encapsulated OVA generated higher frequencies of anti‐tumour H‐2K^b^‐SIINFEKL‐specific CD8^+^ T cells in the blood on day 26 post prime immunisation, as compared to free OVA (~ 10% and 1.5%, respectively) (Figure 3B). Overall, we found that co‐administering CpG with ACM‐OVA was superior in generating H‐2K^b^‐SIINFEKL^+^ CD8^+^ T cells, with significantly higher frequencies of these cells in the blood and inguinal drLN, as compared to mice injected with free OVA, free CpG, ACM‐OVA without CpG or ACM plus Poly(I:C) (Figure 3C).

Since co‐administering ACM‐OVA with CpG increased its T cell priming efficiency, we next tested whether encapsulating CpG within ACM polymersomes would further enhance this effect. The generation of ACM‐CpG has been previously described by us [19]. Here, we immunised C57BL/6 mice with a s.c. injection of either free OVA + free CpG, ACM‐OVA + free CpG or ACM‐OVA + ACM‐CpG or PBS control, followed by a single boost injection of the same formulation after 7 days. Mice subsequently were inoculated s.c. into the right hind flank with 1 × 10^5^ B16‐OVA melanoma cells (day 14 p.i.) and tumour burden and frequency of H‐2K^b^‐SIINFEKL^+^ CD8^+^ T cells was analysed (Figure 3D). Mice immunised with PBS control quickly developed large tumours and succumbed to these within 3 weeks post inoculation, while mice immunised with free OVA + free CpG displayed a delayed but steady tumour growth. In contrast, mice immunised with ACM polymersomes had a drastically delayed and reduced tumour growth. Two out of eight mice in the ACM‐OVA + free CpG group and six out of eight mice in the ACM‐OVA + ACM‐CpG group remained tumour‐free until day 29 when the experiment was ended (Figure 3E). The reduced tumour burden was accompanied by a four‐fold increase in H‐2K^b^‐SIINFEKL^+^ CD8^+^ T cell frequencies in the blood of ACM‐OVA + ACM‐CpG immunised mice on day 20 p.i., as compared to the control groups (Figure 3F). Thus, ACM polymersomes showed promising potential as cancer vaccines, in this prophylactic setting.

We then tested the efficacy of ACM polymersomes in a therapeutic approach. For this purpose, 1 × 10^5^ B16‐OVA melanoma cells were implanted s.c. into the right hind flank of C57BL/6 mice (day 0), followed by a prime injection (day 5) and two boost injections (day 10 and 14) of free OVA + free CpG or ACM‐OVA + free CpG or PBS control. We then analysed the tumour burden, survival and frequency of H‐2K^b^‐SIINFEKL^+^ CD8^+^ T cells in the blood of the mice on day 21 post inoculation (Figure 3G). Overall, injection with ACM‐OVA + free CpG resulted in tumour suppression, with only three out of eight mice eventually developing large tumours > 500 mm^3^ (Figure 3H). We found that tumour growth was delayed, as compared to mice injected with PBS or free OVA + free CpG. This was also reflected by the significantly improved survival of the ACM‐OVA + free CpG group (five out of eight mice versus one out of eight mice for PBS and free OVA + free CpG; Figure 3I). Further, we found that anti‐tumour H‐2K^b^‐SIINFEKL^+^ CD8^+^ T cells were increased by two‐ to three‐fold in the blood of ACM‐OVA + free CpG‐immunised mice on day 21 p.i., as compared to the other two groups (Figures 3J and S4).

Finally, we analysed how long‐lasting these effects would be and re‐challenged mice from the therapeutic or prophylactic ACM‐OVA or ACM‐OVA + ACM‐CpG or ACM‐OVA + free CpG or PBS injection groups with a second round of B16‐OVA inoculation 30 days post initial trial. We found that mice previously subjected to the therapeutic ACM‐injections performed better in controlling tumour growth than PBS treated mice (Figure S4B). and mice that were previously part of the prophylactic ACM‐injection group showed either no or a reduced tumour growth (Figure S4C). Mice from the latter group also showed higher frequencies of OVA+ memory T cells, as compared to PBS injected controls (Figure S4D).

Altogether, immunisation with OVA and CpG delivered by ACM polymersome nanocarriers induced strong anti‐tumour effector CD8^+^ T cell responses, mediated tumour regression and improved the survival of tumour‐bearing mice.

### 2.4 ACM Polymersome Therapy Efficiently Recruits Mononuclear Phagocytes to the Tumour Microenvironment

**FIGURE 4:**
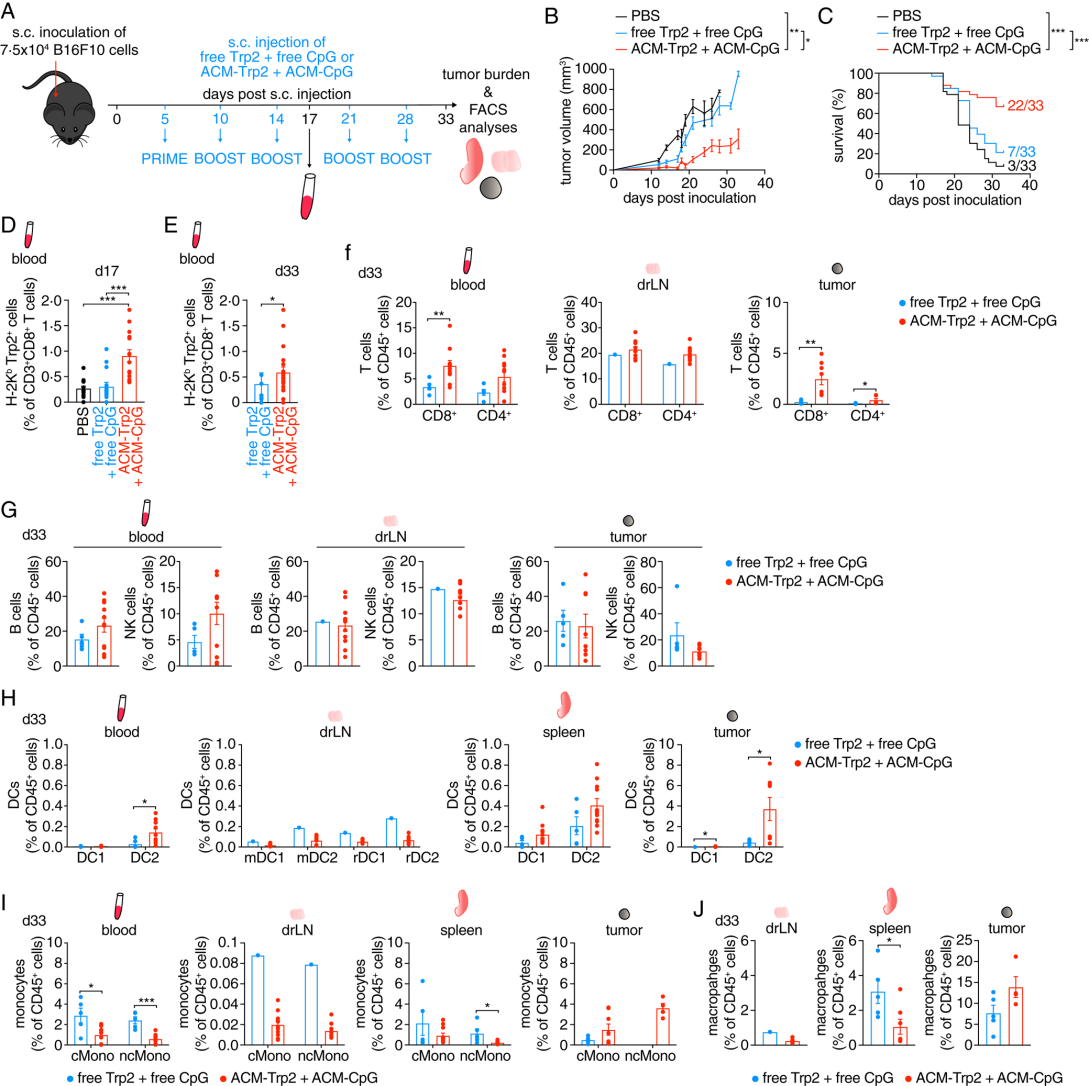
Treatment with ACM‐Trp2 improves survival of B16F10 tumour‐bearing mice. (A) Scheme illustrating prime and boost injection schedule of wildtype mice with free Trp2 peptide plus free CpG (free Trp2 + free CpG) or ACM‐Trp2 plus free CpG (ACM‐Trp2 + free CpG). Mice were inoculated s.c. with 7.5 × 10^4^ B16F10 tumour cells in the right hind flank 5 days prior prime injection (day 0). (B) Graph showing the volume of B16F10 tumours in mice treated with either PBS, free Trp2 + free CpG or ACM‐Trp2 + free CpG. (C) Survival curve of B16F10 tumour‐bearing mice treated with either PBS, free Trp2 + free CpG or ACM‐Trp2 + free CpG. (D) Graph showing the frequency of H‐2K^b^‐Trp2^+^ CD8^+^ T cells in the blood on day 17 post inoculation with B16F10 tumour cells. (E) Graph showing the frequency of H‐2K^b^‐Trp2^+^ CD8^+^ T cells in the blood on day 33 post inoculation with B16F10 tumour cells, at the end of the trial. (F) Graphs showing the frequency of CD8^+^ T cells among CD45^+^ immune cells in the blood, draining LNs (drLN) and tumour on day 33 post inoculation with B16F10 tumour cells. (G) Graphs showing the frequency of B cells and NK cells among CD45^+^ immune cells in the blood, draining LNs (drLN) and tumour on day 33 post inoculation with B16F10 tumour cells. (H) Graphs showing the frequency of different DC subsets among CD45^+^ immune cells in the blood, drLNs, spleen, and tumour on day 33 post inoculation with B16F10 tumour cells. (I) Graphs showing the frequency of monocyte populations among CD45^+^ immune cells in the blood, drLNs, spleen, and tumour on day 33 post inoculation with B16F10 tumour cells. (J) Graphs showing the frequency of macrophages among CD45^+^ immune cells in the drLNs, spleen and tumour on day 33 post inoculation with B16F10 tumour cells. (B–E), Four independent experiments of *n* = 7 to 8 mice each. (G–J) Two independent experiments, *n* = number of mice that survived till day 33 post inoculation. Data are shown as Mean ± SEM. Statistical analysis was done with unpaired two‐tailed *t* test. *p < 0.05; **p < 0.01; ***p < 0.001 and ****p < 0.0001.

We next sought to better understand the immune cell dynamics engaged by ACM polymersome treatment. Here, we opted for the B16F10 melanoma model, which is more aggressive than the B16‐OVA melanoma mouse model. We implanted 7.5 × 10^4^ B16F10 melanoma cells s.c. into the right hind flank of C57BL/6 mice (day 0), followed by one prime (day 5) and four boost injections (day 10, 14, 21, and 28) of either free Trp2 + free CpG or ACM‐Trp2 + ACM‐CpG or PBS (control). Whole blood was drawn and analysed for Trp2‐specific T cells on day 17 post melanoma inoculation. The tumour burden and survival rate were monitored till day 33, when the experiment was ended and tissues were harvested for flow cytometric profiling (Figure 4A). Consistent with our previous data, mice treated with ACM‐Trp2 + ACM‐CpG developed significantly smaller tumours than mice treated with free Trp2 + free CpG or control mice (tumour volume average < 300 mm^3^ vs. > 600 mm^3^, respectively). Three out of 33 mice in the ACM‐Trp2 + ACM‐CpG group even remained tumour‐free until day 33, when the experiment was ended (Figures 4B and S5A). Treatment with ACM‐Trp2 + ACM‐CpG also drastically improved the survival rate of tumour‐bearing mice, 67% as compared to 21% and 9% for mice treated with free Trp2 + free CpG or PBS, respectively (Figure 4C). Similar to our previous trials, this was accompanied by significantly higher frequencies of tumour peptide‐specific CD8^+^ T cells (H‐2K^b^‐Trp2^+^) in the blood of ACM‐Trp2 + ACM‐CpG‐treated mice on day 17 and day 33 p.i., as compared to the other treatment groups (Figure 4D,E). In general, mice treated with ACM‐Trp2 + ACM‐CpG had higher frequencies of CD8^+^ and CD4^+^ T cells in the blood, skin drLN, and tumour as compared to mice treated with free Trp2 + free CpG. This could suggest that encapsulating Trp2 within ACM polymersomes not only improved proliferation of Trp2‐specific effector CD8^+^ T cells but overall stimulated T cell engagement and recruitment to tumours (Figure 4F). Of note, we found no significant differences between T cell populations in the spleen (Figure S5B). However, we noticed a shift from naïve CD8^+^ T cells towards higher frequencies of CD44^hi^CD62L^−^ CD8^+^ effector T cells in the ACM‐Trp2 + ACM‐CpG‐treated mice particularly within the TME (Figure S5C). We also found higher frequencies of B and NK cells in the blood, but not skin drLN nor tumour, of ACM‐Trp2 + ACM‐CpG‐treated mice (Figure 4G).

We also performed an in‐depth analysis of the myeloid cell compartment across tissues and treatment groups. Overall we observed an influx of DC2, but not DC1, in the blood, spleen, and tumours of ACM‐Trp2 + ACM‐CpG‐treated mice on day 33 p.i., with lower cell frequencies across all DC populations in the skin drLN, as compared to mice treated with free Trp2 + free CpG (Figure 4H). Interestingly, this seemed to be accompanied by an increased activation of DCs across tissues, as identified by the upregulation of the co‐stimulatory molecule CD86 on surface of DCs in the skin drLN, spleen, and tumour (Figure S5D). Further, we observed drastic differences in monocyte distribution across tissues between ACM‐Trp2 + ACM‐CpG‐treated versus free Trp2 + free CpG‐treated mice. Frequencies of both, classical and non‐classical, monocytes were vastly increased in the blood and drLN mice treated with free Trp2 + free CpG but basically absent from their tumours. In contrast, in ACM‐Trp2 + ACM‐CpG‐treated mice monocyte frequencies were reduced in blood, skin drLN, and spleen but enriched in the TME (Figure 4I). This was further accompanied by an increase of tumour‐associated macrophages in this treatment group (Figure 4J).

Overall, we found that treatment with ACM‐encapsulated Trp2, rather than free Trp2, significantly improved the survival of tumour‐bearing mice, while aiding tumour regression. This was likely due to enhanced DC activation by ACM‐polymersomes, facilitating efficient priming of tumour‐specific T cells.

### 2.5 ACM Polymersomes Enhance Anti‐PD‐1 Checkpoint Inhibitor Therapy Efficiency

**FIGURE 5:**
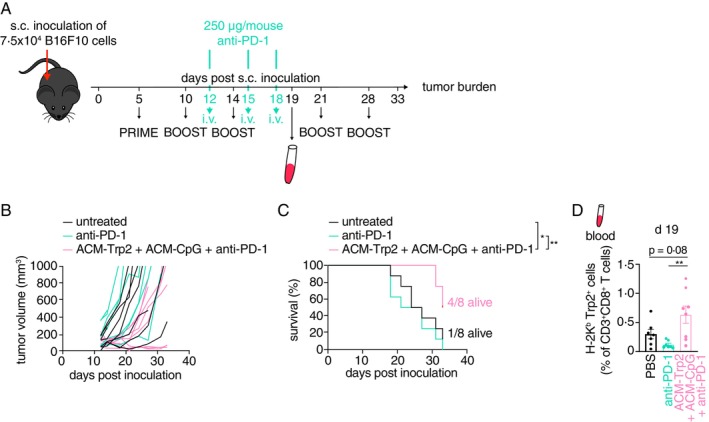
Combination therapy with ACM‐Trp2 and anti‐PD‐1 results in reduced tumour burden further aiding survival of B16F10 tumour‐bearing mice. (A) Graphic illustrating the trial design. Wildtype mice were s.c. inoculated with 7.5 × 10^4^ B16F10 tumour cells in the right hind flank on day 0. Prime and boost s.c. injections of ACM‐Trp2 + ACM‐CpG were given from day 5 on as indicated. In addition, 250 μg of anti‐PD‐1 were injected i.v. on day 12, 15 and 18 post inoculation. (B) Graph showing the volume of B16F10 tumours in untreated mice or mice injected with either anti‐PD‐1 alone or ACM‐Trp2 + ACM‐CpG + anti‐PD‐1. (C) Survival curve of B16F10 tumour‐bearing mice treated with either anti‐PD‐1 alone or ACM‐Trp2 + ACM‐CpG + anti‐PD‐1 or left untreated. (D) Graph showing the frequency of H‐2K^b^‐Trp2^+^ CD8^+^ T cells in the blood on day 19 post inoculation with B16F10 tumour cells. Data are shown as Mean ± SEM. Statistical analysis was done with unpaired two‐tailed *t* test. *p < 0.05; **p < 0.01; ***p < 0.001 and ****p < 0.0001 or as indicted.

Currently, immune checkpoint blockade is a promising therapy option for melanoma patients, with improved survival and overall decent outlook [5, 6, 28]. Programmed cell death‐1 (PD‐1) and its ligand PD‐L1 have been common targets for these therapies, as blockade of PD‐1 enhances anti‐tumour T cell responses and activity [5, 29]. However, depending on disease severity and other factors, PD‐1 inhibition may not lead to the desired long‐term outcome for cancer patients. Thus, we evaluated if combining anti‐PD‐1 therapy with peptide delivery via ACM polymersomes could further improve anti‐tumour immune responses and thus outcome of immune checkpoint therapy. We implanted C57BL/6 mice with 7.5 × 10^4^ B16F10 melanoma cells s.c. into the right hind flank (day 0) and treated them with ACM‐Trp2 + ACM‐CpG on day 5 (prime) and days 10, 14, 21, and 28 (boost). In addition, mice received three doses of 250 μg/mouse anti‐PD‐1 antibody injected intravenously (i.v.) (on days 12, 15, and 18 p.i.). Alternatively, mice were i.v. injected solely with anti‐PD‐1 antibody (on the indicated time points) without ACM polymersomes or left untreated entirely. All mice were analysed for the presences of Trp2‐specific T cells in the blood on day 19 p.i. (Figure 5A). As reported previously [30], mice treated with anti‐PD‐1 antibody barely performed better than untreated mice, in terms of tumour volume and survival (Figure 5B,C). In contrast, mice treated with a combination therapy of ACM‐Trp2 + ACM‐CpG and anti‐PD‐1 showed reduced tumour growth, with almost 40% of mice showing no or only minor tumour growth (Figure 5B). Survival of mice in this group was also vastly improved, as compared to anti‐PD‐1 or untreated mice (4/8 mice as compared to 0/8 and 1/8 of mice respectively) (Figure 5C). This was likely due to the higher frequency of effector tumour‐specific (Trp2^+^) CD8^+^ T cells in the blood of these mice driving tumour regression (Figure 5D). Overall, these data suggested that combining ACM‐peptide polymersome therapy with anti‐PD‐1 therapy further improves anti‐tumour immune responses aiding tumour regression.

### 2.6 Delivery of Peptides Within ACM Polymersomes Boosts Proliferation of Peptide‐Specific Human T Cells

**FIGURE 6:**
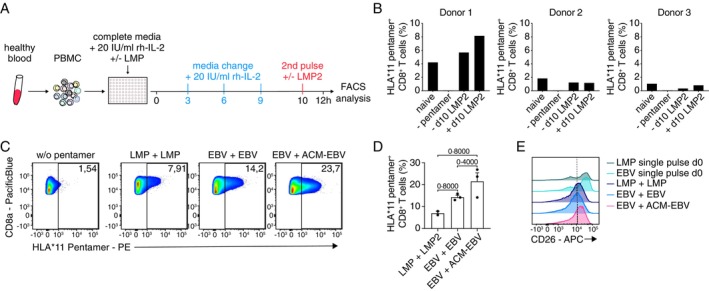
ACM polymersomes boost proliferation of EBV peptide specific T cells. (A) Schematic overview of culture setup to generate peptide specific T cells from primary human blood PBMC in vitro, using LMP and LMP2 peptides. (B) Quantification of the frequency of HLA*11‐specific CD8^+^ T cells among different donors. (C) Exemplary flow cytometry plots and (D) graphs showing frequency of HLA*11‐specific CD8^+^ T cells after stimulation with either LMP peptides, EBV peptides or ACM‐EBV multi‐epitope. (E) Histogram showing the mean fluorescent intensity of CD26 on day 11 of culture on CD8^+^ T cells differentiated with different peptide combinations, as indicated. Data are shown as Mean ± SEM. Statistical analysis was done with unpaired two‐tailed t test. *p < 0.05; **p < 0.01; ***p < 0.001 and ****p < 0.0001 or as indicted.

We previously showed that human CD141^+^ DC1 and CD1c^+^ DC2 from whole blood can efficiently be targeted to uptake ACM‐Rho [19]. Thus, we speculated that uptake of peptide‐loaded polymersomes by human DC would enhance the antigen‐specific T cell response in human, similar to what we had observed in mouse. Here, we ex vivo stimulated human PBMCs with either Epstein–Barr virus (EBV)‐derived peptides or ACM polymersomes carrying multiple EBV‐derived peptides. EBV is an ubiquitous virus linked to increased risk for certain types of cancer [31]. First, we cultured PBMC from three different healthy donors, supplemented with rh‐IL‐2 and stimulated with the EBV‐derived latent membrane protein (LMP) short peptide on day 0, followed by an overnight pulse with the long LMP2 peptide on day 10 (Figure 6A). Among our three donors only donor 1 had notable frequencies of HLA*11/peptide‐specific CD8^+^ T cells to begin with, with increased proliferation of these post LMP/LMP2 stimulation, suggesting donors 2 and 3 likely had not been exposed to EBV before (Figure 6B). We then tested whether encapsulation of a newly synthesised multi‐EBV peptide construct into ACM polymersomes (ACM‐EBV) could further enhance proliferation and frequencies of HLA*11/EBV‐peptide specific CD8^+^ T cells. Indeed, we found that PBMCs stimulated with EBV‐peptide and pulsed with ACM‐EBV on day 10 of culture, had vastly increased frequencies of HLA*11/peptide‐specific CD8^+^ T cells, as compared to the other stimulation groups, for example > 20% for EBV/ACM‐EBV as compared to < 10% for LMP/LMP2 stimulation/pulse (Figure 6C,D).

CD26^hi^CD8^+^ T cells have been described as a population of early effector memory cells [32, 33], and have been discussed as drivers of anti‐tumour immunity [34]. We observed that CD8^+^ T cells in this culture setting had high expression levels of CD26 after single dose of peptide stimulation, though these seemed to be downregulated slightly after the day 10 pulse. However, pulsing with ACM‐EBV did increase CD26 expression levels on CD8^+^ T cells, in comparison to T cells that were pulsed with LMP/LMP2 or EBV/EBV instead (Figure 6E).

Overall, we found ACM polymersomes to be promising new nanocarriers with the ability to enhance and improve the generation and effect of peptide‐specific T cells, offering new possibilities for vaccination and therapy strategies of cancer patients.

## 3 Discussion

Here, we demonstrated that self‐assembling ACM polymersomes are efficient nanocarriers for cancer‐derived peptides and other cargo. We found that delivering antigens within these was superior in eliciting strong innate and adaptive immune responses, as compared to delivering free antigens. We observed improved immune activation, tumour regression, and survival of ACM‐immunised mice in murine models of B16‐OVA and B16F10 melanoma.

Despite recent accomplishments in personalised medicine and the introduction of immune checkpoint inhibitor therapies for cancer patients [1, 2, 4, 5], there remains a high demand for new accessible and safe therapies and vaccines that can induce long‐lasting, potent immune responses and immune memory against cancer. Further, these new strategies also need to overcome tumour‐induced immunosuppression. Designing cancer vaccines and therapies comes with a set of hurdles, from the sheer variety of cancers, organs affected, early versus late‐stage cancers to metastasizing tumours and beyond. In recent years, several approaches have been made to develop biocompatible, yet stable, drug delivery systems, for example in the shape of lipid‐based carriers. However, while liposomes are more accessible to immune cells, they are rather unstable risking the pre‐mature release of their cargo and thus insufficient immune activation [20]. Other new options for vaccine design have become available also, including synthetic peptide vaccines, that allow for more targeted immune activation. Thus, we hypothesized that packaging and delivering peptides within biocompatible, yet stable membrane‐like vesicles should improve peptide uptake by phagocytosing and antigen‐presenting cells, thus enhance their overall immunogenicity in a safe manner.

Safety of such artificial cell membrane‐like vaccines has been confirmed by us in a previous study, where ACM polymersomes encapsulating SARS‐CoV‐2 spike protein and CpG adjuvant were administered intramuscularly (i.m.) or intranasally (i.n.) to New Zealand white rabbits in a repeated‐dose, Good Laboratory Practice toxicological study (Lam et al., 2022). This ACM COVID‐19 vaccine was well tolerated via each route of administration and rabbits did not present with adverse clinical signs, mortality, local reactions nor systemic toxicity. Increased cellular infiltrate and inflammation at the injection site along with increased cellularity of the muscle‐draining iliac lymph node were interpreted as local responses to vaccination. This vaccine was subsequently evaluated in a recently completed phase 1 clinical trial (ClinicalTrials.gov identifier: NCT05385991) and found to be well tolerated after both, i.m. or i.n., administration. Local reactions and systemic events were generally mild to moderate and transient.

Further, administration of two doses of ACM‐encapsulated SARS‐CoV‐2 spike protein and CpG adjuvant subcutaneously or intramuscular resulted in robust spike‐specific serum IgG and live virus neutralising titers and spike‐reactive splenic CD4^+^ and CD8^+^ T cells that responded with IFNγ production were detected 40 days after boost, indicating a durable, memory response.

Here, we encapsulated OVA and Trp2 peptides within our previously developed self‐assembling ACM polymersomes, and indeed found that this peptide delivery strategy increased immunogenicity of tumour‐peptides in mice.

Though ACM‐peptide delivery itself resulted in APC activation and generation of peptide‐specific T cells, we wondered if we could even further enhance its efficiency. Especially since immunotherapy resistance in cancer patients is a consistent problem that needs to be overcome [35]. Tumour cells have been shown to be able to modulate the TME to their advantage, thus most therapies now aim at increasing local inflammation in the TME. Here, we tried to achieve this by either co‐injecting free CpG or CpG encapsulated within ACM polymersomes with ACM‐peptides during immunisation of tumour‐bearing mice. CpG binding to TLR9 on DC results in their activation and maturation, generally accompanied by secretion of pro‐inflammatory chemokines and cytokines, such as I interferon (IFN‐I). This aids antigen‐presentation by DC to naïve T cells and generation of antigen‐specific effector CD8^+^ T cells, which is crucial for anti‐tumour immunity. Indeed, we observed an increased DC activation, as measured by the upregulation of the co‐stimulatory molecule CD86, post immunisation with ACM‐Trp2 + free CpG, aiding the efficient priming of Trp2‐tumour‐specific T cells. IFN‐I itself was shown to directly and indirectly act on other immune cells, driving immune responses but also to directly impact tumour cells. Here its role has been rather ambiguous, with some reports stating that acute exposure of tumour cells to high levels of IFN‐I result in growth arrest and cell death, and others stating that chronic exposure to low levels of IFN‐I might aid tumour survival and immune‐evasion Further, secretion of IFN‐I was shown to also lead to increased concentrations of CXCL9 and CXCL10 within the TME, CCL3 and CCL5 in the spleen, promoting the recruitment of NK and CD8^+^ T cells to these sites [26, 36, 37]. Indeed, we found increased frequencies of CD8^+^ T cells, peptide‐specific CD8^+^ T cells, and early memory T cells in tumours of mice immunised with ACM‐peptide + free CpG. However, as constant low exposure to IFN‐I might be benefiting tumours to actually evade the immune system, there is a fine balance that needs to be kept in how much we want to engage IFN‐I responses using ACM polymersome + CpG based cancer vaccines or therapies. Thus, future experiments should focus on investigating IFN‐I responses engaged by ACM polymersomes.

In general, ACM polymersomes are a modular platform, meaning the architecture of these polymersomes can be formed using just the amphiphilic block copolymers or a polymer‐lipid hybrid system. We can dope different amounts of lipids in these polymersomes, which helps to improve the electrostatic interaction between the polymersomes and the modality that is being encapsulated, further improving the loading of these modalities. This freedom to dope different lipids with the amphiphilic block copolymers to form assemblies allows for effective encapsulation of a variety of different modalities. We here demonstrated that different payloads could be delivered using ACM polymersomes, such as peptides (here Trp2), proteins (here OVA), and oligonucleotides (here CpG). Further, ACM polymersomes could be used to deliver a wider range of antigens or adjuvants or DNA, mRNA, and different small molecules, in combination with some lipids. However, the pharmacokinetic and pharmacodynamic for these novel polymers yet needs to be understood in greater detail and in the context of different tissues and vaccine types and will be subject of future studies.

Overall, we observed increased frequencies of Rho^+^ LCs and macrophages within the drLN after s.c. injection of ACM‐Rho, suggesting that ACM polymersomes taken up by phagocytotic cells can efficiently be shuttled to sites of T cell priming. We also found that ACM‐Rho polymersomes were maintained long‐term in the subcutaneous compartment, as compared to free Rho. This could provide a constant pool of low‐key immunisation and might be beneficial for generating a stable immune memory, while avoiding over‐activation of MNPs and T cells. However, macrophages were the predominant cell type labelled by ACM‐Rho polymersomes, in line with earlier studies showing that dermal macrophages can phagocytose tattoo ink and retain the it within vacuoles in their cytoplasm, thus are responsible for tattoos to last within the skin [38]. Future experiments should aim at clarifying whether the overserved persistence of s.c. Rho is really due to maintenance of an ACM‐Rho pool, or to macrophages simply storing Rho long‐term.

Anti‐PD‐1 immune checkpoint inhibitor therapy has proven promising and with generally good outcome for cancer patients [1, 5]. PD‐1 in combination with its ligand PD‐L1 dampens effector T cell activity by disrupting downstream signalling of the T cell receptor, thus has a strong immunosuppressive function. PD‐L1 often is overexpressed on the surface of tumour cells, which then can actively evade immune responses by engaging with PD‐1 on the surface of T cells, resulting in tumour progression [39]. Checkpoint inhibitor therapy makes use of this mechanism by actively blocking this T cell inhibition, thus restoring effector T cell function and mediating anti‐tumour immune responses [1, 2, 4, 5]. However, there are certain limitations with checkpoint inhibitor therapies that need to be overcome, such as unresponsiveness of some patients to the treatment, recurrence of tumours post treatment, side effects of the treatment, which can include autoimmune responses, and others. We found, that combining anti‐PD‐1 therapy with ACM‐peptide polymersome therapy might help overcome some of these, by more targeted immune activation and that immunisation with a combination therapy improved generation of tumour‐specific CD8^+^ T cells and tumour regression. Thus, ACM polymersomes offer a pipeline to new approaches to help improve the shortcomings of current immune therapies. Thus, future experiments will aim at co‐administering multiple drugs or adjuvants within the same ACM polymersome formulation, and cell‐specific targeting of ACM‐peptide polymersomes to DC to specifically harness these cells own immune capacities, allowing for targeted, controlled and specific immune activation in cancer therapy.

## 4 Materials and Methods

### 4.1 Preparation of ACM‐OVA, ACM‐Trp2 and ACM‐CpG

ACM‐OVA was prepared by the thin‐film rehydration method, followed by extrusion. A 95.2 mg of PEG_13_‐*b*‐PBD_22_ polymer in chloroform was mixed with 5.9 mg DOTAP in chloroform at a mol ratio of 85:15. Subsequently, chloroform was removed by rotary evaporator followed by drying for 1 h at high vacuum. An 8 mL solution of 400 μg/mL OVA was added and the solution stirred at 600–700 rpm in the dark for 5 h at 4°C. The resultant solution was extruded 21 times through a 200 nm Nuclepore hydrophilic polycarbonate membrane filter using a 1 mL mini‐extruder (Avanti Polar Lipids). Non‐encapsulated OVA protein was removed by dialysis over 2 days using 300 kDa molecular weight cut‐off (MWCO) cellulose ester membrane (Spectrum Laboratories Inc.) against 1X PBS at 4°C with one buffer exchange. Encapsulation of OVA protein was quantified by densiometric analysis using a known OVA protein standards in Fiji ImageJ software (v. 1.52a). ACM‐Trp2 was prepared by the solvent dispersion method, followed by extrusion. A 380 mg/mL stock solution of DOTAP and PEG_13_‐*b*‐PBD_22_ polymer were prepared by dissolving solid DOTAP and polymer in tetrahydrofuran (THF). 0.15 equivalent (1.05 μmol) of DOTAP stock solution and 0.85 equivalent (5.95 μmol) of polymer stock solution were mixed and vortexed to prepare Solution A. This was added slowly to 10 mL of Solution B (400 μg/mL of Trp2 peptide) while constantly mixing (600–700 rpm) at room temperature. The resultant solution was extruded 21 times through a 200 nm membrane filter (Avanti Polar Lipids) using a 1 mL mini‐extruder (Avanti Polar Lipids). Non‐encapsulated peptide was removed by overnight dialysis against 1X PBS with three buffer exchanges. Encapsulation of Trp2 peptide were quantified by Agilent ZORBAX 300SB‐C8 HPLC column using known Trp2 peptide standards. ACM‐CpG was prepared by solvent dispersion method, followed by extrusion. The polymer lipid mixture was made by dissolving and mixing 0.15 equivalent (1.05 μmol) of DOTAP stock solution and 0.85 equivalent (5.95 μmol) of PEG_13_‐*b*‐PBD_22_ in THF. This mixture was added to 3 mL of buffer solution containing 200 μg/mL of CpG‐1826 while constantly mixing (600–700 rpm) at room temperature. The resultant solution was extruded 21 times through a 200 nm membrane filter (Avanti Polar Lipids) using a 1 mL mini‐extruder (Avanti Polar Lipids). Non‐encapsulated CpG was removed by overnight dialysis against 1X PBS.

### 4.2 Preparation of ACM‐Rho and ACM‐DQ‐OVA

ACM‐Rho and ACM‐DQ‐OVA were made by thin‐film rehydration, followed by rehydration. For ACM‐Rho, a dried polymer film comprises of PEG_13_‐b‐PBD_22_ polymer and Rhodamine B‐terminated PEG_13_‐b‐PBD_22_ with ratio of 99:1 w/v was rehydrated with buffer. The suspension was stirred overnight at 4°C, followed by extrusion using a 1 mL mini‐extruder (Avanti Polar Lipids) and 200 nm membrane filter (Avanti Polar Lipids). The excess polymers were removed by overnight dialysis using a 300 kDa MWCO cellulose ester membrane (Spectrum Laboratories Inc) against 1 × PBS at 4°C. The ACM‐DQ‐OVA was prepared by rehydrating 57 mg of PEG_13_‐b‐PBD_22_ polymer with 3 mL of 100 μg/mL DQ‐OVA solution overnight at 4°C. The resultant solution was extruded 21 times through a 200 nm Nuclepore hydrophilic polycarbonate membrane filter using a 1 mL mini‐extruder (Avanti Polar Lipids). Non‐encapsulated DQ‐OVA protein was removed by dialysis over 2 days using 300 kDa molecular weight cut‐off (MWCO) cellulose ester membrane (Spectrum Laboratories Inc.) against 1X PBS at 4°C with one buffer exchange.

### 4.3 Mice

C57BL/6J mice were obtained from In Vivos Pte Ltd. Mice were maintained in the A*STAR Biological Resource Centre (BRC) animal facility before use at 6–12 weeks of age. Only female mice were used for the B16‐OVA and B16F10 melanoma trials. All experiments and procedures were approved by the Institutional Animal Care and Use Committee of the Biological Resource Center (Agency for Science, Technology and Research, Singapore) in accordance with the guidelines of the Agri‐Food and Veterinary Authority and the National Advisory Committee for Laboratory Animal Research of Singapore (IACUC No. 181357, 211651).

### 4.4 B16‐OVA and B16F10 Melanoma Mouse Models and Treatments

The murine melanoma cell lines B16‐OVA and B16F10 were cultured and maintained in vitro in DMEM (4.5 g/L glucose) with L‐glutamine plus 10% fetal bovine serum (FBS) and 1% penicillin/streptomycin. Mice were inoculated subcutaneously in the right hind flank with 1 × 10^5^ B16‐OVA melanoma cells or 7.5 × 10^4^ B16F10 melanoma cells and tumour growth was monitored until the end of experiment. Mice were euthanized when their tumour volume exceeded 1000 mm^3^, when they reached a moribund state, or when their tumours became necrotic. Free CpG and ACM‐CpG doses at 5 μg CpG/mouse were administered as stated. Free OVA and ACM‐OVA at 10 μg OVA/mouse, and free Trp2 peptide and ACM‐Trp2 at 18 μg Trp2 peptide/mouse were also given as described. Mouse anti‐PD‐1 (Clone RMP1‐14) was purchased from BioXcell and administered as described.

### 4.5 Tissue Preparation and Data Analysis for Flow Cytometry

Blood was collected from the facial vein or orbital plexus directly into PBS + 10 mM EDTA, skin, spleen, drLN, and tumour samples were harvest and stored in PBS on ice till further use. Spleen, drLN, and tumour were transferred into RPMI containing DNaseI and collagenase, disrupted using tweezers, and digested for 30 min at 37°C. Digest was stopped by adding PBS + 10 mM EDTA and cell suspensions were transferred into a fresh tube over a 70 μm nylon mesh sieve. Red blood cells then were lysed using RBC lysis buffer (eBioscience), and single cell suspensions were passed through a 70 μm nylon mesh sieve before further use. Skin samples were incubated in RPMI containing 4 U/mL Dispase, for 1 h at 37°C. Skin samples then were transferred into RPMI containing DNaseI and collagenase, minced, and digested for 1 h at 37°C. Digest was stopped by adding PBS + 10 mM EDTA, cell suspensions were transferred into a fresh tube over a 70 μm nylon mesh sieve to obtain single cell suspensions. Cells were then used for flow cytometry analysis. Antibodies for flow cytometry were purchased from BD, Biolegend, or eBioscience (for details see Table S2). For multi‐parameter analysis, non‐specific antibody binding was prevented by pre‐incubating cells with either purified anti‐CD16/32 (Fc‐block, clone 2.4G2, BD Biosciences) or n‐rat and n‐mouse serum (both from Sigma). Lymphoid and myeloid cells were identified by labelling with fluorochrome‐ or biotin‐conjugated monoclonal antibodies (mAbs) to mouse (for details see Table S2). Peptide specific T cells were analysed by staining with PE‐conjugated H2‐kb‐SIINFEKL Dextramer (Immudex, Cat: JD02163) and PE‐conjugated H2‐kb Trp2 peptide (SVYDFFVWL) Pentamer (Immudex, Cat: JD02199) were used to stain for tumour‐peptide‐specific CD8^+^ T cells. Flow cytometry acquisition was performed on a 5‐laser BD LSR II (BD) or a BD FACSymphony using FACSDiva software, and data subsequently analysed with FlowJo software (Tree Star).

### 4.6 In Vitro T Cell Differentiation

PBMC from the blood of healthy donors were cultured in complete media for 10 days in the presence of 20 IU/mL rh‐IL‐2 with or without peptide. Media was changed every 3 days and PBMC were pulse with a second peptide dose on day 10 of culture. HLA*11 pentamer positive T cells were analysed 12 h after the second peptide pulse by flow cytometry.

### 4.7 In Vitro Treatment of Human PBMCs With ACM Formulations

PBMCs from two to five healthy donors were seeded at 0.3 to 1 million cells per well of a 96‐well U‐bottom plate in 0.2 mL of complete RPMI. 15 μg/mL of ACM‐CpG or empty polymersomes were incubated with PBMCs for 2 days at 37°C, 5% CO_2_. Cytokines secreted in culture supernatant were evaluated using the LEGENDplex COVID‐19 Cytokine Storm Panel 1 (BioLegend). ACM‐Rho and ACM‐Cy5 of similar stock concentrations (based on particle derived count rates) were added at 2‐fold serial dilutions (1:10 to 1:160) alone or in combination for 3 h. Cells were washed and stained with eFluor 455 UV fixable viability dye (Thermo Fisher Scientific) at 1:400 in PBS for 30 min at 4°C. Fc receptors were blocked using Human TruStain FcX reagent (BioLegend) for 10 min at 4°C. Cell surface markers were stained with the following antibodies for 30 min at 4°C: BUV395‐CD45 (BD Biosciences, Cat: 563792), BV510‐CD56 (BioLegend, Cat: 318340), PE‐Cy7‐CD3 (BioLegend, Cat:300316), BUV737‐CD19 (BD Biosciences, Cat: 612756), PerCP‐Cy5.5‐CD14 (BioLegend, Cat: 301824), BV785‐CD16 (BioLegend, Cat: 302046), APC‐Cy7‐HLA‐DR (BioLegend, Cat: 307618), BV650‐CD123 (BioLegend, Cat: 306020) and FITC‐CD11c (Thermo Fisher Scientific, Cat: 11‐0116‐42). Data acquisition is described above.

### 4.8 Statistical Analysis

Statistical analyses were done using Prism software (Graphpad). Comparison between two groups was done using unpaired two‐tailed *t* test, with Mann–Whitney test and for Kaplan–Meier survival curves the Log‐rank (Mantel‐Cox) test was performed. Or as indicated in the corresponding figure legends. *p* values < 0.05 were considered as statistically significant.

## Author Contributions

R.J.D., W.W.H., V.H., F.M.D. and F.G. designed experiments. R.J.D., W.W.H., V.H., G.S., F.M.D., J.S. and G.R. performed experiments. R.J.D., W.W.H., V.H., F.M.D. and F.G. analysed data. J.H.L., A.K.K., T.A.C., T.W.C., S.V. and M.N. prepared and provided crucial reagents. M.N. provided intellectual guidance. R.J.D. and F.G. wrote the paper with editing input from all authors. F.G. conceptualised the study.

## Conflicts of Interest

F.M.D., J.H.L., G.S., A.K.K., T.A.C., T.W.C., S.V. and M.N. were or are employees of ACM Biolabs Pte Ltd., working on the commercialization of the ACM platform. The authors have no additional financial interests.

## Supporting information


**Figure S1.** ACM preparation methods. (A) Thin film method for polymersome preparation and modality encapsulation: (a) Polymer and lipids are dried to prepare an uniform thin film of the lipids. (b) The thin film is rehydrated with buffer and modality to be encapsulated. (c) The rehydrated film and encapsulated modality are extruded using a 0.2 μm membrane to make polymersomes of uniform size. (d) the extruded samples are dialyzed to remove the unencapsulated modalities. (B) Co‐solvent method for polymersome preparation and modality encapsulation: (a) Modality to be encapsulated in prepared in buffer solution at the required concentration. (b) The polymer lipid stock is slowly added to the buffer solution while the buffer is vortexed continuously. The thin film is rehydrated with buffer and modality to be encapsulated. (c) the buffer solution is extruded using a 0.2 μm membrane to make polymersomes of uniform size. (d) the extruded samples are dialyzed to remove the unencapsulated modalities. (C) Cytokine production by human PBMCs treated with ACM‐CpG or empty ACM polymersomes. PBMCs from five healthy donors were treated for two days with 15 μg/mL of encapsulated CpG or a matched amount of empty polymersomes. Cytokines secreted in culture supernatant were evaluated using the LEGENDplex™ COVID‐19 Cytokine Storm Panel 1 (BioLegend). Welch’s *t* test was performed. ** *p* ≤ 0.01; *** *p* ≤ 0.001.

**Figure S2.** ACM‐supplied Rhodamine is maintained subcutaneously providing long‐term stimulation of MNP. (A) Images showing the persistence of s.c. injected free Rho vs. ACM‐Rho from one to 140 days post injection, in comparison to the PBS control. (B) Pie charts showing the distribution of myeloid cell populations among the Rho^+^ fraction on the indicated days p.i. of ACM‐Rho. (C) FACS plots showing Rho‐signals in distinct MNP populations in the blood and spleen and tumor on day 6 post s.c. injection. (D) Graphs showing the frequency of Rho/DQ^+^ MNP in the skin and inguinal drLN over time. (E, F) Graphs showing the distribution of myeloid cell populations among the Rho^+^ fraction on the indicated days post s.c. or i.m injection of ACM‐Rho in the skin (E) or inguinal LN (F). Data are shown as Mean ± SEM. Statistical analysis was done with unpaired two‐tailed *t* test. *p < 0.05; **p < 0.01; ***p < 0.001 and ****p < 0.0001.

**Figure S3.** Ex vivo uptake of ACM polymersomes by human PBMCs. PBMCs were treated with either ACM‐Rho or ACM‐Cy5 or in a 1:1 combination of ACM‐Rho plus ACM‐Cy5 for three hours. Uptake of polymersomes was evaluated by flow cytometry. (A) Gating strategy. (B) Co‐uptake of ACM‐Rho and ACM‐Cy5 by monocytes and DCs.

**Figure S4.** ACM‐OVA plus CpG leads to expansion of H‐2K^b^‐SIINFEKL‐specific CD8^+^ T in vaccinated and therapeutically injected mice. (A) FACS plots showing H‐2K^b^‐SIINFEKL MHCI‐peptide complex^+^ CD8^+^ T cells in the blood on day 21 post s.c. inoculation of B16‐OVA cells. (B) Tumor growth upon rechallenge with B16‐OVA 30 post end of initial therapeutic trial and B16‐OVA challenge. (C) Tumor growth upon rechallenge with B16‐OVA 30 post end of initial prophylactic trial and B16‐OVA challenge. (D) OVA^+^ and OVA^‐^ memory T cells in the blood of tumor‐bearing or control mice 30 days after the rechallenge.

**Figure S5.** ACM‐Trp2 + ACM‐CpG treatment leads to expansion of B16F10 tumour specific T cells and reduced tumour growth. (A) Graph showing volume of B16F10 tumors in individual mice treated with either PBS, free Trp2 + free CpG or ACM‐Trp2 + ACM‐CpG. Shown are single graphs for four independent trials. (B) Graphs showing the frequency of CD4^+^ and CD8^+^ T cells among CD45^+^ immune cells in the spleen on day 33 post inoculation with B16F10 tumor cells. (C) Graphs showing the frequency of naïve, memory and effector CD8^+^ T cell populations among CD45^+^ immune cells in the blood, drLN and tumor on day 33 post inoculation with B16F10 tumor cells. (D) Graphs showing the mean fluorescent intensity of the activation marker CD86 on DC populations in the drLN, spleen and tumor on day 33 post inoculation with B16F10 tumor cells. Data are shown as Mean ± SEM. Statistical analysis was done with unpaired two‐tailed *t* test. *p < 0.05; **p < 0.01; ***p < 0.001 and ****p < 0.0001.

**Table S1.** Physical characterisation of the ACM formulations.

**Table S2.** Antibodies used for flow cytometry were purchased from BD, Biolegend or eBioscience and details for each antibody used are listed in the table.

## Data Availability

Datasets associated with the current study are available from the corresponding author upon reasonable request.
