# Supplementary figures and images for: A Novel Polymersome Nanocarrier Promotes Anti‐Tumour Immunity by Improved Priming of CD8 + T Cells
Source: Immunology. 2025 Jan 28;175(1):21–35. doi: 10.1111/imm.13903 (PMC11982605; doi:10.1111/imm.13903)

**A**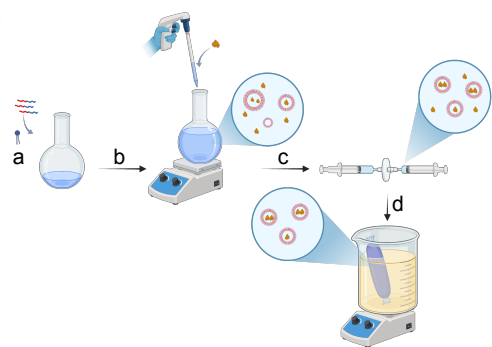**B**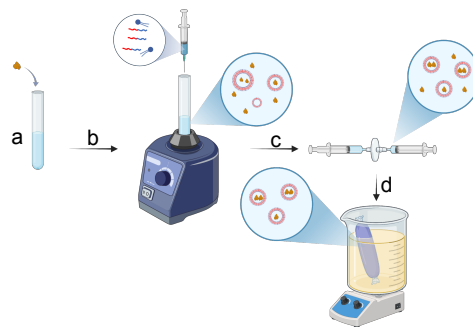**C**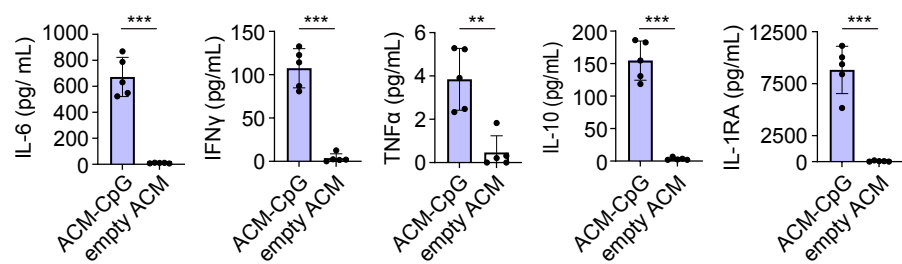

Supplement: Supplementary file 1 — Figure S1. ACM preparation methods. (A) Thin film method for polymersome preparation and modality encapsulation: (a) Polymer and lipids are dried to prepare an uniform thin film of the lipids. (b) The thin film is rehydrated with buffer and modality to be encapsulated. (c) The rehydrated film and encapsulated modality are extruded using a 0.2 μm membrane to make polymersomes of uniform size. (d) the extruded samples are dialyzed to remove the unencapsulated modalities. (B) Co‐solvent method for polymersome preparation and modality encapsulation: (a) Modality to be encapsulated in prepared in buffer solution at the required concentration. (b) The polymer lipid stock is slowly added to the buffer solution while the buffer is vortexed continuously. The thin film is rehydrated with buffer and modality to be encapsulated. (c) the buffer solution is extruded using a 0.2 μm membrane to make polymersomes of uniform size. (d) the extruded samples are dialyzed to remove the unencapsulated modalities. (C) Cytokine production by human PBMCs treated with ACM‐CpG or empty ACM polymersomes. PBMCs from five healthy donors were treated for two days with 15 μg/mL of encapsulated CpG or a matched amount of empty polymersomes. Cytokines secreted in culture supernatant were evaluated using the LEGENDplex™ COVID‐19 Cytokine Storm Panel 1 (BioLegend). Welch’s t test was performed. ** p ≤ 0.01; *** p ≤ 0.001. [file IMM-175-21-s003.pdf]

**A**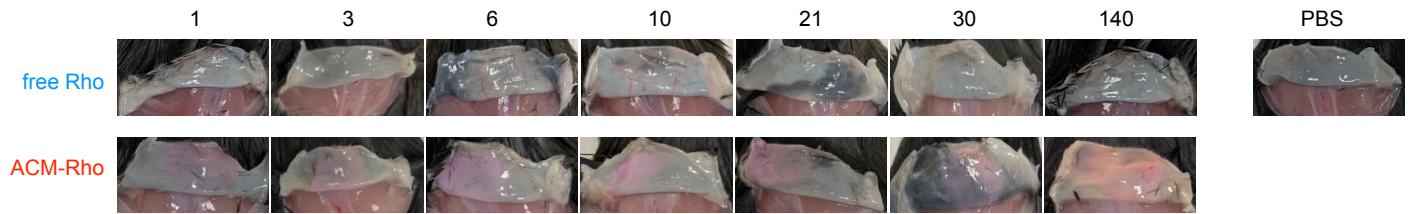**B**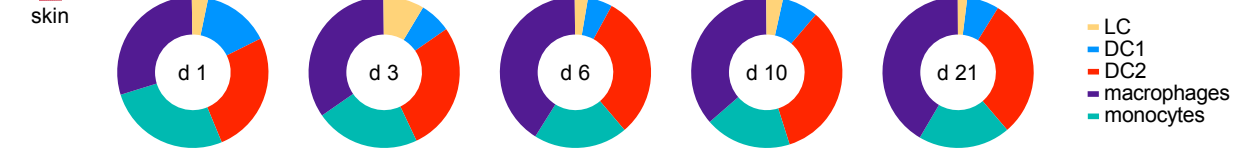**C**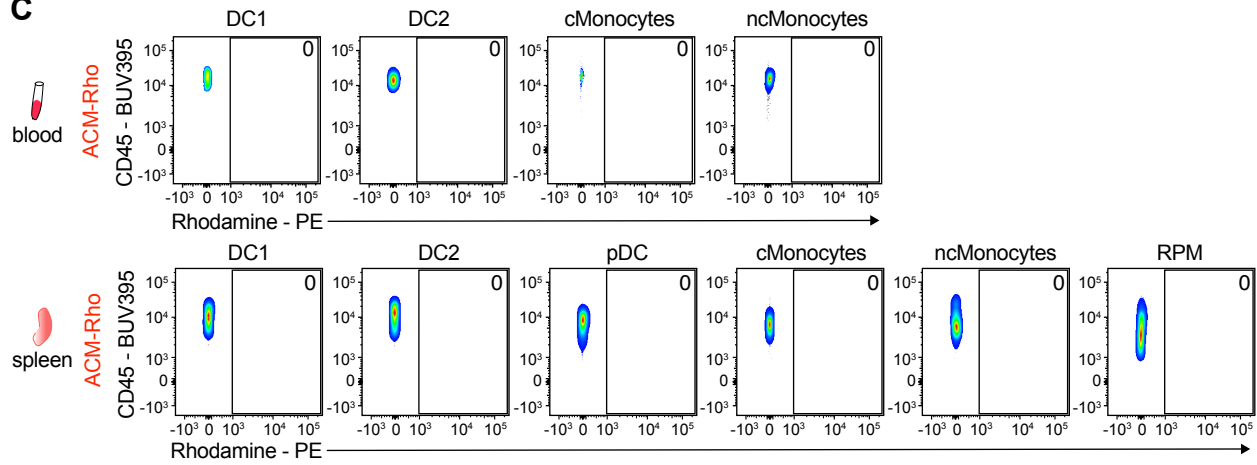**D**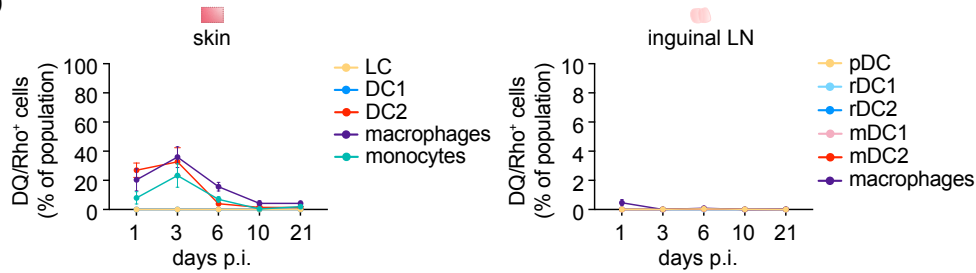**E**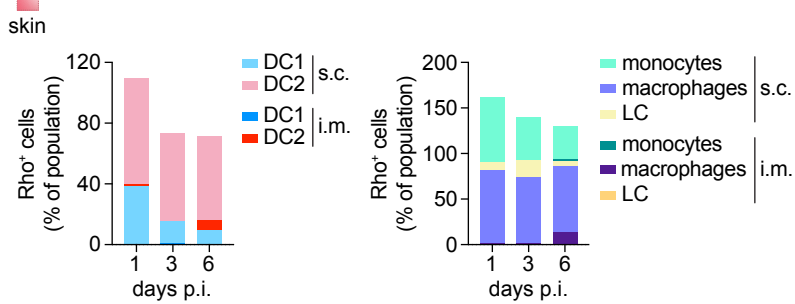**F**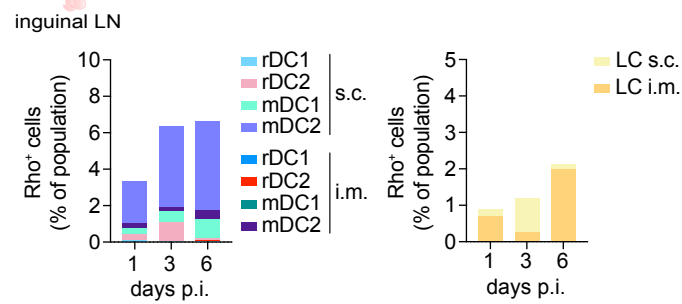

Supplement: Supplementary file 2 — Figure S2. ACM‐supplied Rhodamine is maintained subcutaneously providing long‐term stimulation of MNP. (A) Images showing the persistence of s.c. injected free Rho vs. ACM‐Rho from one to 140 days post injection, in comparison to the PBS control. (B) Pie charts showing the distribution of myeloid cell populations among the Rho+ fraction on the indicated days p.i. of ACM‐Rho. (C) FACS plots showing Rho‐signals in distinct MNP populations in the blood and spleen and tumor on day 6 post s.c. injection. (D) Graphs showing the frequency of Rho/DQ+ MNP in the skin and inguinal drLN over time. (E, F) Graphs showing the distribution of myeloid cell populations among the Rho+ fraction on the indicated days post s.c. or i.m injection of ACM‐Rho in the skin (E) or inguinal LN (F). Data are shown as Mean ± SEM. Statistical analysis was done with unpaired two‐tailed t test. *p < 0.05; **p < 0.01; ***p < 0.001 and ****p < 0.0001. [file IMM-175-21-s002.pdf]

**A**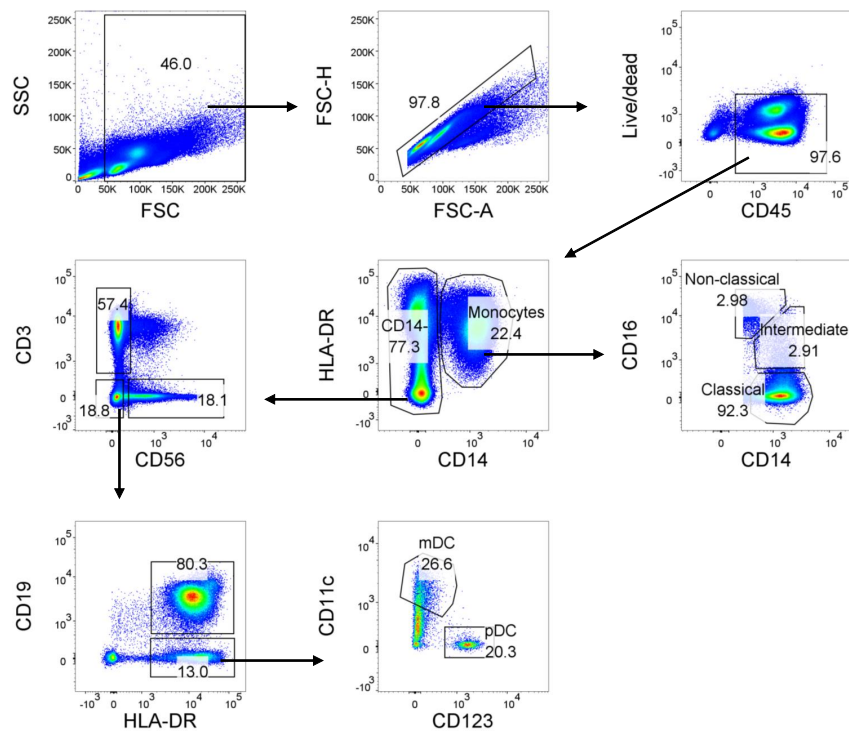**B**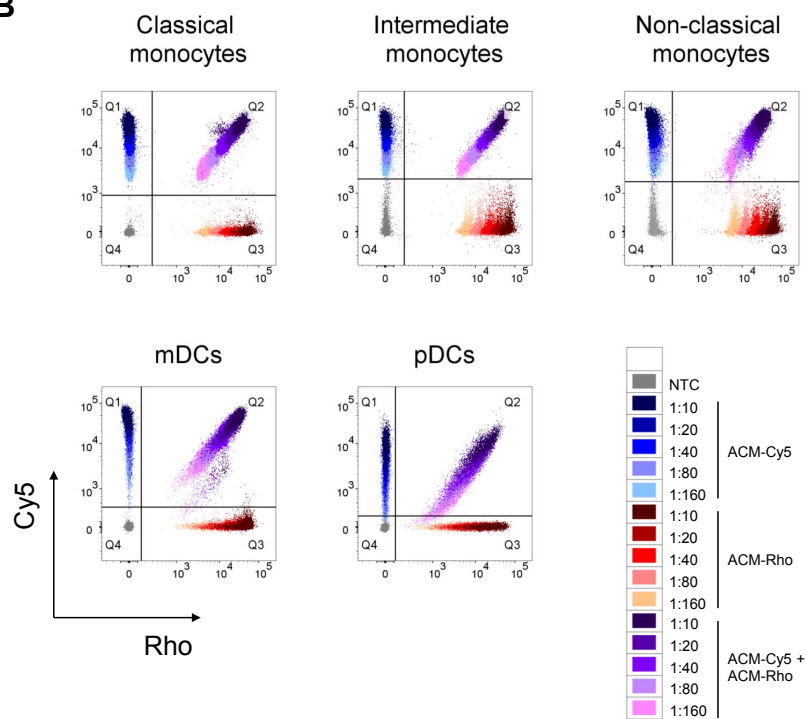

Supplement: Supplementary file 3 — Figure S3. Ex vivo uptake of ACM polymersomes by human PBMCs. PBMCs were treated with either ACM‐Rho or ACM‐Cy5 or in a 1:1 combination of ACM‐Rho plus ACM‐Cy5 for three hours. Uptake of polymersomes was evaluated by flow cytometry. (A) Gating strategy. (B) Co‐uptake of ACM‐Rho and ACM‐Cy5 by monocytes and DCs. [file IMM-175-21-s006.pdf]

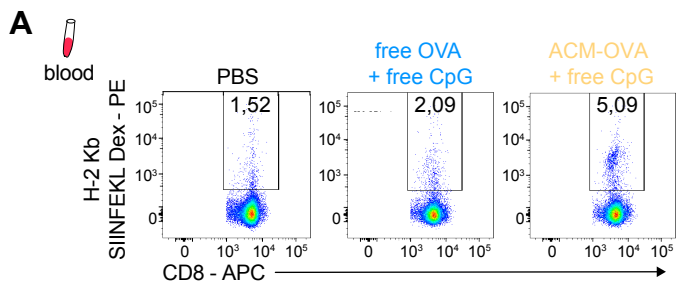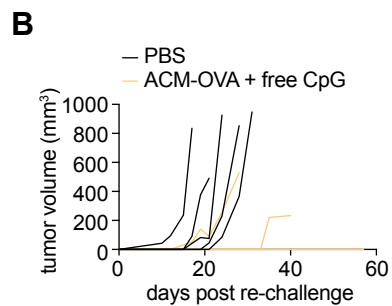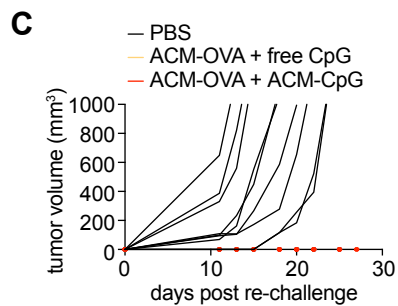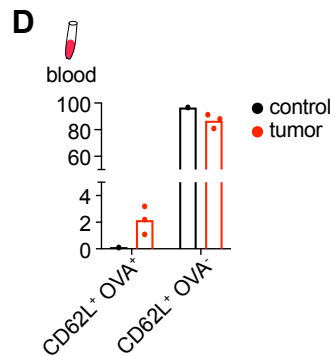

Supplement: Supplementary file 4 — Figure S4. ACM‐OVA plus CpG leads to expansion of H‐2Kb‐SIINFEKL‐specific CD8+ T in vaccinated and therapeutically injected mice. (A) FACS plots showing H‐2Kb‐SIINFEKL MHCI‐peptide complex+ CD8+ T cells in the blood on day 21 post s.c. inoculation of B16‐OVA cells. (B) Tumor growth upon rechallenge with B16‐OVA 30 post end of initial therapeutic trial and B16‐OVA challenge. (C) Tumor growth upon rechallenge with B16‐OVA 30 post end of initial prophylactic trial and B16‐OVA challenge. (D) OVA+ and OVA‐ memory T cells in the blood of tumor‐bearing or control mice 30 days after the rechallenge. [file IMM-175-21-s001.pdf]

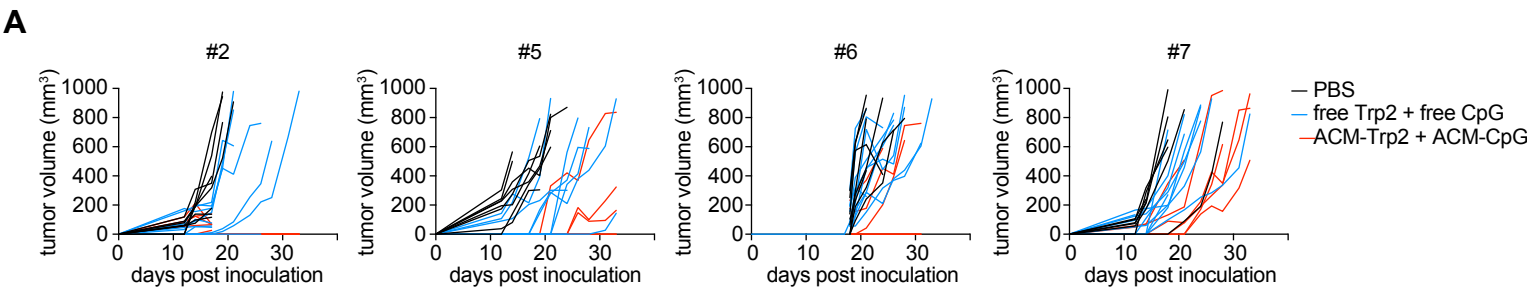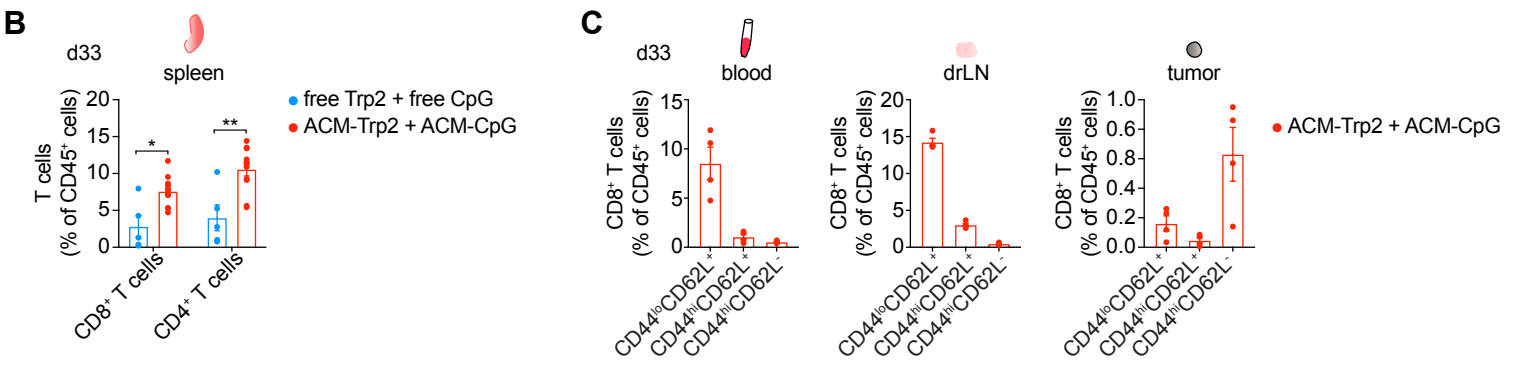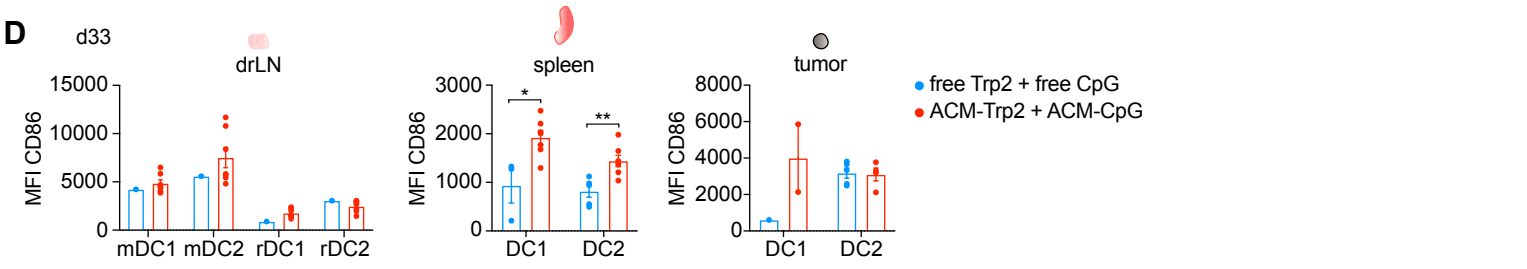

Supplement: Supplementary file 5 — Figure S5. ACM‐Trp2 + ACM‐CpG treatment leads to expansion of B16F10 tumour specific T cells and reduced tumour growth. (A) Graph showing volume of B16F10 tumors in individual mice treated with either PBS, free Trp2 + free CpG or ACM‐Trp2 + ACM‐CpG. Shown are single graphs for four independent trials. (B) Graphs showing the frequency of CD4+ and CD8+ T cells among CD45+ immune cells in the spleen on day 33 post inoculation with B16F10 tumor cells. (C) Graphs showing the frequency of naïve, memory and effector CD8+ T cell populations among CD45+ immune cells in the blood, drLN and tumor on day 33 post inoculation with B16F10 tumor cells. (D) Graphs showing the mean fluorescent intensity of the activation marker CD86 on DC populations in the drLN, spleen and tumor on day 33 post inoculation with B16F10 tumor cells. Data are shown as Mean ± SEM. Statistical analysis was done with unpaired two‐tailed t test. *p < 0.05; **p < 0.01; ***p < 0.001 and ****p < 0.0001. [file IMM-175-21-s004.pdf]
