# Supplementary material for: A Novel Polymersome Nanocarrier Promotes Anti‐Tumour Immunity by Improved Priming of CD8 + T Cells
Source: Immunology. 2025 Jan 28;175(1):21–35. doi: 10.1111/imm.13903 (PMC11982605; doi:10.1111/imm.13903)
Supplement: Supplementary file 6 — Table S1. Physical characterisation of the ACM formulations. [file IMM-175-21-s007.docx]

| **Formulation** | **API** | **Method** | **Size (nm)** | **PDI** | **Zeta potential (mV)** |
| --- | --- | --- | --- | --- | --- |
| ACM-OVA | protein | thin film rehydration | 162.0 | 0.160 | 0.0 |
| ACM-Trp2 | peptide | co-solvent | 129.9 | 0.149 | 23.5 |
| ACM-CpG | oligodeoxy-nucleotide | co-solvent | 116.7 | 0.166 | 21.2 |
| ACM-Rho | fluorescent dye | thin film rehydration | 168.9 | 0.127 | 26.4 |
| ACM-Cy5 | fluorescent dye | thin film rehydration | 168.3 | 0.123 | 26.3 |
| ACM-DQ-OVA | fluorogenic substrate for proteases | thin film rehydration | 222.5 | 0.178 | 0.0 |

**Supplementary Table 1.** Physical characterization of the here used ACM formulations.
