# Supplementary material for: A Novel Polymersome Nanocarrier Promotes Anti‐Tumour Immunity by Improved Priming of CD8 + T Cells
Source: Immunology. 2025 Jan 28;175(1):21–35. doi: 10.1111/imm.13903 (PMC11982605; doi:10.1111/imm.13903)
Supplement: Supplementary file 7 — Table S2. Antibodies used for flow cytometry were purchased from BD, Biolegend or eBioscience and details for each antibody used are listed in the table. [file IMM-175-21-s005.docx]

﻿

| **Antibody** | **Fluochrome** | **Clone** | **Manufacturer** | **Catalogue number** | **Concentration used** |
| --- | --- | --- | --- | --- | --- |
| Anti-mouse CD3 | PerCP-Cy5.5 | 17A2 | eBioscience | 46-0032-82 | 1:200 |
| Anti-mouse CD3 | BV510 | 500A2 | BD Biosciences | 740113 | 1:200 |
| Anti-mouse CD4 | BUV605 | 100548 | BioLegend | RM4-5 | 1:200 |
| Anti-mouse CD4 | PE-Cy7 | 25-0042-82 | eBioscience | RM4-5 | 1:200 |
| Anti-mouse CD8a | APC | 53-6.7 | BD Biosciences | 553035 | 1:200 |
| Anti-mouse CD8a | eFluor 450 | 53-6.7 | eBioscience | 48-0081-82 | 1:200 |
| Anti-mouse CD8a | PE-CF594 | 53-6.7 | BD Pharmingen | 562283 | 1:200 |
| Anti-mouse CD11b | BV650 | M1/70 | BioLegend | 101259 | 1:200 |
| Anti-mouse CD11c | BV605 | N418 | BioLegend | 117334 | 1:200 |
| Anti-mouse CD11c | PE-Cy7 | N418 | BioLegend | 117318 | 1:200 |
| Anti-mouse CD16/32 | Pure | 2.4G2 | BD Pharmingen | 553142 | 1:50 |
| Anti-mouse CD19 | BV510 | 1D3 | BD Horizon | 562956 | 1:200 |
| Anti-mouse CD19 | Pacific Blue | 1D3 | eBioscience | 57-0193-82 | 1:200 |
| Anti-mouse CD44 | APC-eFluor 780 | IM7 | eBioscience | 47-0441-82 | 1:200 |
| Anti-mouse CD45 | BUV395 | 30-F11 | BD Biosciences | 564279 | 1:200 |
| Anti-mouse CD45R/B220 | Alexa Fluor 700 | RA3-6B2 | eBioscience | 56-0452-82 | 1:200 |
| Anti-mouse CD49b | BV510 | HMα2 | BD Biosciences | 740133 | 1:200 |
| Anti-mouse CD62L | Biotin | MEL-14 | BioLegend | 104403 | 1:200 |
| Anti-mouse CD64 | BV711 | X54-5/7.1 | BioLegend | 139311 | 1:200 |
| Anti-mouse CD86 | APC | GL1 | eBioscience | 17-0862-82 | 1:200 |
| Anti-mouse CD103 | APC | 2E7 | eBioscience | 17-1031-82 | 1:200 |
| Anti-mouse CD103 | Biotin | 2E7 | eBioscience | 13-1031-82 | 1:200 |
| Anti-mouse EpCAM | BUV605 | G8.8 | BioLegend | 118227 | 1:200 |
| Anti-mouse F4/80 | PE/Dazzle 594 | BM8 | BioLegend | 123146 | 1:200 |
| Anti-mouse Ly6C | APC-Cy7 | HK1.4 | BioLegend | 128026 | 1:200 |
| Anti-mouse Ly6C | PerCP-Cy5.5 | HK1.4 | eBioscience | 45-5932-82 | 1:200 |
| Anti-mouse Ly6G | BV510 | 1A8 | BD Biosciences | 740157 | 1:200 |
| Anti-mouse MHCII | Alexa Fluor 700 | M5/114.15.2 | BioLegend | 107622 | 1:200 |
| Anti-mouse MHCII | BV785 | M5/114.15.2 | BioLegend | 107645 | 1:200 |
| Anti-mouse SiglecH | PerCP-Cy5.5 | 551 | BioLegend | 129614 | 1:200 |
| Anti-human CD1c | PE-Cy7 | L161 | BioLegend | 331516 | 1:100 |
| Anti-human CD3 | BUV650 | SP34-2 | BD Bioscience | 563916 | 1:100 |
| Anti-human CD3 | PerCP-Cy5.5 | OKT3 | BioLegend | 317336 | 1:100 |
| Anti-human CD4 | BUV737 | SK3 | BD Horizon | 564306 | 1:100 |
| Anti-human CD8a | BV421 | RPA-T8 | BD Bioscience | 562428 | 1:100 |
| Anti-human CD11c | BUV395 | 7G3 | BD Bioscience | 563787 | 1:100 |
| Anti-human CD14 | Alexa Fluor 700 | M5E2 | BD Bioscience | 557923 | 1:100 |
| Anti-human CD14 | BV605 | M5E2 | BioLegend | 301834 | 1:100 |
| Anti-human CD16 | BV650 | 3G8 | BD Bioscience | 563692 | 1:100 |
| Anti-human CD16 | FITC | CB16 | eBioscience | 11-0168-42 | 1:100 |
| Anti-human CD19 | APC-Cy7 | MB19-1 | abcam | ab51533 | 1:50 |
| Anti-human CD19 | PerCP/Cy5.5 | HIB19 | BioLegend | 302230 | 1:100 |
| Anti-human CD26 | APC | BA5b | BioLegend | 302710 | 1:100 |
| Anti-human CD38 | Biotin | HIT2 | BioLegend | 303518 | 1:100 |
| Anti-human CD45 | V500 | Hi30 | BD Horizon | 560777 | 1:100 |
| Anti-human CD45 | ECD | J33 | Beckman Coulter | IM2710U | 1:100 |
| Anti-human CD107a | FITC | H4A3 | BioLegend | 328605 | 1:100 |
| Anti-human HLA-DR | BUV785 | L243 | BioLegend | 307642 | 1:100 |
| H2-kb-SIINFEKL | PE |  | Immudex | JD02163 | 5 μl/sample |
| H2-kb Trp2 peptide (SVYDFFVWL) | PE |  | Immudex | JD02199 | 5 μl/sample |
| Streptavidin | BUV737 |  | BD Horizon | 564293 | 1:300 |
| Streptavidin | PE-CF594 |  | BD Biosciences | 562284 | 1:300 |
